# Monophosphorylation of cardiac troponin-I at Ser-23/24 is sufficient to regulate cardiac myofibrillar Ca^2+^ sensitivity and calpain-induced proteolysis

**DOI:** 10.1074/jbc.RA117.001292

**Published:** 2018-04-18

**Authors:** Abel Martin-Garrido, Brandon J. Biesiadecki, Hussam E. Salhi, Yasin Shaifta, Cristobal G. dos Remedios, Serife Ayaz-Guner, Wenxuan Cai, Ying Ge, Metin Avkiran, Jonathan C. Kentish

**Affiliations:** From the ‡ King's College London British Heart Foundation Centre of Excellence, School of Cardiovascular Medicine and Sciences, London SE1 7EH, United Kingdom,; the § Department of Physiology and Cell Biology, Davis Heart and Lung Research Institute, Ohio State University, Columbus, Ohio 43210,; the ¶ Bosch Institute, Discipline of Anatomy and Histology, University of Sydney, New South Wales 2006, Australia, and; the Departments of ‖ Cell and Regenerative Biology and; §§ Chemistry,; ** Molecular and Cellular Pharmacology Training Program, and; ‡‡ Human Proteomics Program, University of Wisconsin, Madison, Wisconsin 53705

**Keywords:** troponin, phosphorylation, protein kinase D (PKD), calpain, cardiac muscle, cardiomyocyte, Ca^2+^ sensitivity, myofibril, sarcomere

## Abstract

The acceleration of myocardial relaxation produced by β-adrenoreceptor stimulation is mediated in part by protein kinase A (PKA)-mediated phosphorylation of cardiac troponin-I (cTnI), which decreases myofibrillar Ca^2+^ sensitivity. Previous evidence suggests that phosphorylation of both Ser-23 and Ser-24 in cTnI is required for this Ca^2+^ desensitization. PKA-mediated phosphorylation also partially protects cTnI from proteolysis by calpain. Here we report that protein kinase D (PKD) phosphorylates only one serine of cTnI Ser-23/24. To explore the functional consequences of this monophosphorylation, we examined the Ca^2+^ sensitivity of force production and susceptibility of cTnI to calpain-mediated proteolysis when Ser-23/24 of cTnI in mouse cardiac myofibrils was nonphosphorylated, mono-phosphorylated, or bisphosphorylated (using sequential incubations in λ-phosphatase, PKD, and PKA, respectively). Phos-tag gels, Western blotting, and high-resolution MS revealed that PKD produced >90% monophosphorylation of cTnI, primarily at Ser-24, whereas PKA led to cTnI bisphosphorylation exclusively. PKD markedly decreased the Ca^2+^ sensitivity of force production in detergent-permeabilized ventricular trabeculae, whereas subsequent incubation with PKA produced only a small further fall of Ca^2+^ sensitivity. Unlike PKD, PKA also substantially phosphorylated myosin-binding protein-C and significantly accelerated cross-bridge kinetics (*k*_tr_). After phosphorylation by PKD or PKA, cTnI in isolated myofibrils was partially protected from calpain-mediated degradation. We conclude that cTnI monophosphorylation at Ser-23/24 decreases myofibrillar Ca^2+^ sensitivity and partially protects cTnI from calpain-induced proteolysis. In healthy cardiomyocytes, the basal monophosphorylation of cTnI may help tonically regulate myofibrillar Ca^2+^ sensitivity.

## Introduction

Although cytosolic Ca^2+^ is the primary regulator of myofibril contractile activity in cardiac cells, post-translational modifications of sarcomeric proteins play a major additional role in modulating the Ca^2+^ sensitivity and dynamic behavior of myofibrils (1). For example, during stimulation of the cardiac β_1_-adrenoreceptor–G_s_–adenylyl cyclase–cAMP signaling cascade, protein kinase A (PKA)^4^ phosphorylates cardiac troponin-I (cTnI), thereby increasing the rate of Ca^2+^ dissociation from troponin-C (2) and decreasing myofibrillar Ca sensitivity. Together with abbreviation of the intracellular Ca^2+^ transient, these cTnI-mediated effects contribute to the acceleration of cardiac relaxation (lusitropic effect) during β-adrenoreceptor stimulation and may also contribute to the positive inotropic effect (3, 4). Additionally, PKA-induced phosphorylation of cardiac myosin–binding protein C (cMyBP-C) accelerates myosin cross-bridge kinetics, which likely contributes to the inotropic and lusitropic effects. It is well established that the phosphorylation of cTnI by PKA occurs at both Ser-23 and Ser-24 (Ser-22 and Ser-23 if the initiating methionine is not counted) in the N terminus of cTnI, and previous evidence suggests that phosphorylation of *both* serines is required for the depression of Ca^2+^ sensitivity (5, 6). With cMyBP-C, the PKA-induced increase in cross-bridge kinetics is achieved by phosphorylation of serines 273, 282, and 302 in the M domain, although single-site phosphorylation of Ser-282 by RSK (p90 ribosomal S6 kinase) (7) or Ser-302 by protein kinase D (PKD) (8) can have a similar effect.

PKA-mediated phosphorylation of cTnI and cMyBP-C also regulates contractile function indirectly by reducing the susceptibility of these proteins to proteolysis. The Ca^2+^-activated protease calpain has been variously suggested to be responsible for the cTnI C-terminal cleavage observed during ischemia/reperfusion (I/R) (9), which may contribute to stunning (9, 10), or for the N-terminal cleavage that has been seen in chronic simulated microgravity (11), heart failure (12), and even in healthy hearts (11). Phosphorylation of cTnI *in vitro* by PKA has been found to slow the calpain-mediated degradation of cTnI (13) (although at which cleavage site remains unclear) and therefore could have a protective role in limiting cTnI contractile dysfunction under these various conditions. Similarly, cleavage of cMyBP-C in the N terminus has been found during I/R, leading to the formation of a poison peptide C0C2f fragment that inhibits contractile function, and this cleavage is inhibited by pseudophosphorylation of serines 273, 282, and 302 (14, 15).

In a previous study, we showed that PKD, an end effector of the G_q_–phospholipase C–diacylglycerol+inositol 1,4,5-trisphosphate (InsP_3_) cascade, could mimic the functional effects of PKA by decreasing myofibrillar Ca^2+^ sensitivity via phosphorylation of cTnI Ser-23/24 and increasing cross-bridge kinetics by phosphorylation of cMyBP-C at Ser-302 (8). We assumed that the actions of PKD on cTnI were, like PKA, due to bisphosphorylation at Ser-23/24. However, we now find that PKD phosphorylates only one of the Ser-23/24 sites. If this was also true in our previous study (8), it would indicate that *mono*phosphorylation at Ser-23/24 can regulate myofibrillar Ca^2+^ sensitivity, contradicting the prevailing view (5, 6). To investigate this possibility, we produced myofibril preparations in which cTnI in the intact sarcomere is present with Ser-23/24 uniformly unphosphorylated, mono-phosphorylated (using PKD), or bisphosphorylated (using PKA) and compared the effects on myofibrillar contractile performance and on the protection of cTnI from calpain-mediated proteolysis. An essential part of this study was the development of a novel dephosphorylation protocol to remove pre-existing phosphorylation from the Ser-23/24 sites. Our data demonstrate that monophosphorylation of cTnI at Ser-23/24 (predominantly Ser-24) regulates the Ca^2+^ sensitivity of cardiac myofibrils and partially protects cTnI from calpain-induced proteolysis.

## Results

### Phosphorylation of cTnI by PKD or PKA in cardiac myofibrils

**Figure 1.**
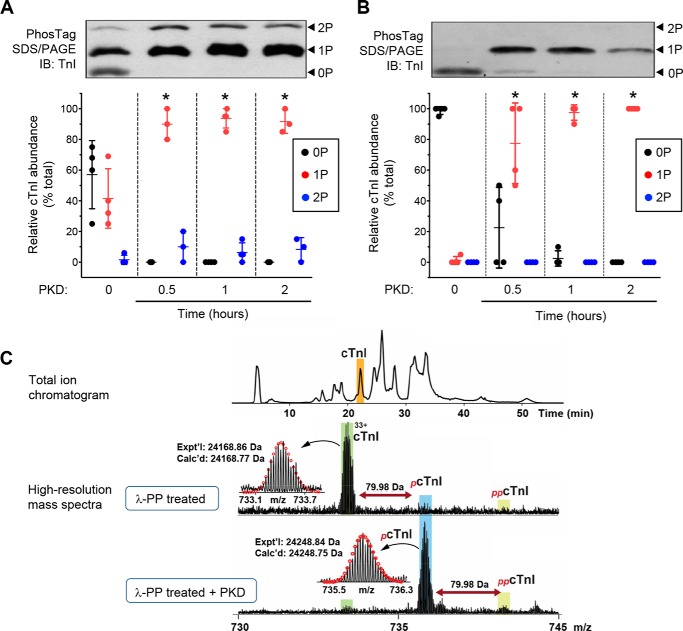
**PKD monophosphorylates cTnI in the sarcomere.** *A*, mouse cardiac myofibrils were incubated in PKD for different times, separated in SDS-PAGE with Phos-tag reagent, and blotted with a specific antibody against cTnI. *Top panel*, representative Western blot. *Bottom panel*, relative abundance of the three bands of cTnI in each lane. *Symbols* show the data from individual experiments; *vertical bars* show the mean ± S.D. (*n* = 3 experiments; *, *p* < 0.05 *versus* nontreated). *IB*, immunoblot. *B*, as *A*, except isolated myofibrils were incubated with λ-PP before incubation in PKD (*n* = 4; *, *p* < 0.05 *versus* nontreated). Note that incubation with PKD produces monophosphorylation of cTnI almost exclusively. *C*, top-down high-resolution MS analysis of cTnI from mouse myofibrils. *Top panel*, representative total ion chromatogram showing separation and elution of cTnI. *Bottom panel*, high-resolution mass spectra of cTnI from λ-PP–treated myofibrils before and after 1-h incubation of the myofibrils with PKD. *Horizontal arrows* indicate the position of unphosphorylated (*cTnI*), mono-phosphorylated (*_p_cTnI*), and bisphosphorylated cTnI (*_pp_cTnI*). *Insets*, isotopically resolved molecular ions with the calculated most abundant molecular weight (*Calc'd*) based on the amino acid sequence and experimental most abundant molecular weight (*Expt'l*). *Circles* represent the theoretical isotopic abundance distribution of the isotopomer peaks corresponding to the assigned mass.

We demonstrated previously that PKD phosphorylates cTnI at Ser-23/24 (and cMyBP-C at Ser-302) in isolated myofibrils (7, 8, 16). In our previous studies, cTnI phosphorylation was assessed using a pSer-23/24 antibody, but in this study, we used SDS-PAGE with Phos-tag^TM^ reagent to quantify the different phosphorylation states of cTnI (Phos-tag binds to phosphate groups and retards protein migration according to the number of phosphorylated residues on the protein). As Fig. 1 illustrates, Phos-tag gels showed three different phosphorylation states of cTnI, which we assigned on the basis that PKA is known to produce bisphosphorylation of cTnI in the sarcomere. Even before the addition of any kinase, there was basal phosphorylation of cTnI in these myofibrils, even though the mice had received a β-adrenoreceptor antagonist (Fig. 1*A*), with about 40% present as the monophosphorylated form (1P) and about 60% as the unphosphorylated form (0P). Unexpectedly, after incubation of the myofibrils with constitutively active PKD, the proportion of cTnI that was monophosphorylated increased to more than 90%, but there was no statistically significant increase in the proportion of the bisphosphorylated (2P) form. Because the cTnI was basally phosphorylated before kinase addition, the effects of PKD incubation on cTnI could not be unambiguously interpreted. We therefore dephosphorylated all subsequent myofibril preparations using λ phosphatase (λ-PP) prior to PKD incubation. This protocol (which included additional steps to make it also suitable for use with skinned trabeculae) removed all visible endogenous phosphorylation from cTnI (Fig. 1*B*). Subsequent incubation with PKD induced exclusively mono-phosphorylation of cTnI, which was complete after 1 h of incubation. No statistically significant bisphosphorylation of TnI was observed even after 2 h of PKD incubation.

As a check of the validity of these results, cTnI phosphorylation was assessed using a different technique: high-resolution MS (Fig. 1*C*). In the λ-PP–treated myofibrils, the proportions of un-, mono-, and bisphosphorylated forms of cTnI were 88%:10%:2%, respectively. After incubation of the myofibrils in PKD, the pattern changed to 5%:92%:3%, confirming that incubation of the myofibrils in PKD almost exclusively resulted in monophosphorylation of cTnI.

**Figure 2.**
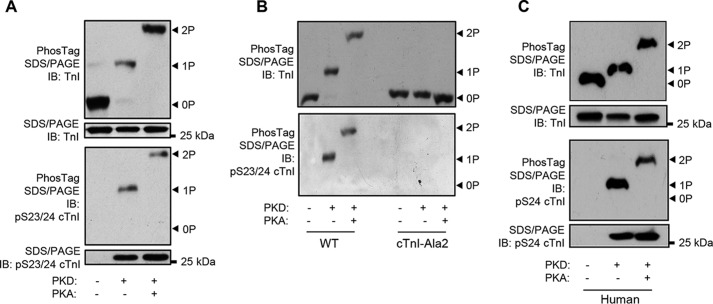
**Determination of the cTnI site targeted by PKD.** *A*, Phos-tag gels of mouse λ-PP–treated myofibrils after incubation in PKD alone (1 h) or PKD (1 h) followed by PKA (1 h). Western blots of Phos-tag gels used specific antibodies against total TnI or pSer-23/24 TnI. *IB*, immunoblot. *B*, comparison of results using myofibrils from WT mice or cTnI-Ala2 transgenic mice. Other conditions were as for *A. C*, Western blots using human λ-PP–treated cardiac myofibrils incubated with PKD alone or PKD followed by PKA and then probed for TnI or pSer-24 cTnI. Other conditions were as for *A*. All gels are representative of at least three experiments.

The above results provide no information about the cTnI amino acid residue(s) targeted by PKD. To examine whether the relevant cTnI site was one (or either) of the Ser-23/24 residues, λ-PP–treated myofibrils were incubated first with PKD and then with PKA. As shown in Fig. 2*A* and Fig. S1, PKD induced monophosphorylation of cTnI, whereas PKA induced bisphosphorylation. There was no evidence for a trisphosphorylated state of cTnI, indicating that the preferred target for PKD was one of the “PKA” sites, *i.e.* Ser-23 or Ser-24. In support of this, an antibody against pSer-23/24 cTnI recognized the PKD-induced cTnI phosphorylation (Fig. 2*A*, *bottom panel*). Furthermore, myofibrils from transgenic cTnI-Ala2 mice (in which Ser-23 and Ser-24 are mutated to nonphosphorylatable alanine residues) remained unphosphorylated after PKD or PKA incubations, confirming the identity of the phospho-acceptor residue(s) that are selectively targeted by these kinases (Fig. 2*B*). We conclude that PKD monophosphorylation occurs at the Ser-23/24 sites in cTnI. It may be noted that the phosphoserine 23/24 cTnI antibody was not selective for the bisphosphorylated form of the protein but detected both the mono- and bisphosphorylated forms (Fig. 2, *A* and *B*), as reported previously (17).

To interrogate whether PKD targets Ser-23 or Ser-24, we used a phosphorylation-specific antibody (mAb14) that has been shown to selectively bind to pSer-24 in human cTnI (17). This antibody did not recognize mouse phosphorylated cTnI,^5^ so we repeated the PKD/PKA incubations using λ-PP–treated myofibrils prepared from human donor heart samples (Fig. 2*C*). PKD induced monophosphorylation of cTnI in human myofibrils as in mouse myofibrils. There was substantial labeling of both the mono- and bisphosphorylated forms of human cTnI by the mAb14 antibody. Because there must be full phosphorylation of Ser-24 in the bisphosphorylated samples, the apparent similarity in the Ser-24 labeling intensities in the PKD-treated and PKA-treated myofibrils would suggest that most, or possibly all, of the monophosphorylation of PKD-treated cTnI is at Ser-24.

Autoradiography using [^32^P]ATP was performed to investigate whether other myofibrillar proteins were significantly phosphorylated by PKD (and PKA). The autoradiogram (Fig. S2) indicates that cTnI and, to a lesser extent, cMyBP-C were the major myofilament proteins phosphorylated by either kinase.

To investigate the functional consequences of cTnI monophosphorylation, we used the above dephosphorylation/phosphorylation protocol (Fig. 2) to produce sarcomeric cTnI in the unphosphorylated, monophosphorylated, and bisphosphorylated forms and compared the effects on two factors known to be influenced by cTnI phosphorylation: myofibrillar contractile performance and the protection of cTnI from calpain-mediated proteolysis.

### Effects of PKD or PKA on myofibrillar Ca^2+^ sensitivity and cross-bridge kinetics

**Figure 3.**
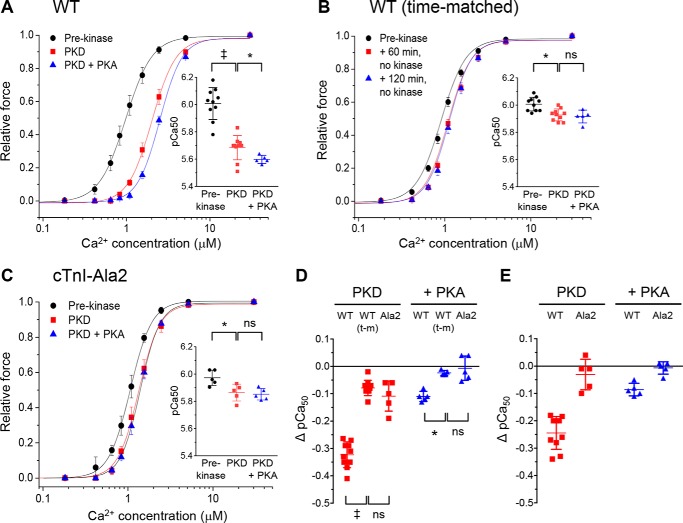
**Comparison of the effects of PKD and PKA on the force–Ca^2+^ relationship of mouse skinned trabeculae.** *A*, forces were measured in λ-PP–treated skinned muscles before (*Pre-kinase*) and after incubations in PKD (*PKD*) and then PKA (*PKD*+*PKA*). Forces expressed relative to that at 30 μmol/liter Ca^2+^. *Inset*, mean *p*Ca_50_ (−log[Ca^2+^]) values. *Symbols* show the data from individual experiments; *vertical bars* show the mean ± S.D. *B*, WT time-matched controls (*no kinase*) corresponding to each incubation in *A. C*, experiments as for *A* but using skinned muscles from cTnI-Ala2 mice. *D*, changes in Ca^2+^ sensitivity (Δ*pCa*_50_) produced by PKD with or without PKA compared with WT time-matched controls. Data labeled *PKD* show the difference in *p*Ca_50_ from the corresponding prekinase *p*Ca_50_. Data labeled +*PKA* show the difference in *p*Ca_50_ from the corresponding PKD *p*Ca_50_. *WT(t-m)* are the time-matched controls (as in *B*). *E*, the same data as in *D* but after subtraction of the mean Δ*p*Ca_50_ of the corresponding time-matched controls to illustrate the time-independent effects of the kinases. For all panels, *n* = 10 for the pre-kinase and PKD incubation groups in WT muscles, and *n* = 5 for all other groups. ‡, *p* < 0.0001; *, *p* < 0.05; *ns*, nonsignificant (all paired *t* tests, *n* = 5 or *n* = 10).

Myofibrillar Ca^2+^ sensitivity and cross-bridge kinetics (*k*_tr_) were measured in λ-PP–treated skinned trabeculae before and after PKD incubation and then again after incubation in PKA (Fig. 3 and Table S1). Time-matched controls were included (no kinases) because pilot experiments showed that there was a small but statistically significant decrease in Ca^2+^ sensitivity over the first hour or so of the experiment (Fig. 3*B*). The reason for this is unclear, but it may be related to a time-dependent reduction in titin-based passive force, which would decrease myofibrillar Ca^2+^ sensitivity (18). Incubation in PKD produced a large decrease of Ca^2+^ sensitivity, with the *p*Ca_50_ (*p*Ca for 50% activation of force) falling from 6.00 ± 0.04 to 5.68 ± 0.03 (mean ± S.E., *n* = 10), a mean reduction of 0.32 ± 0.01 (Fig. 3*D*). In contrast, the subsequent incubation in PKA (performed in five experiments) produced only a small further decrease in *p*Ca_50_ from 5.70 ± 0.01 to 5.59 ± 0.01 (*n* = 5), an additional decrease of 0.11 ± 0.01. If we subtract the mean *p*Ca_50_ changes in the corresponding time-matched controls, then the time-independent fall in *p*Ca_50_ is 0.24 ± 0.03 for PKD (Fig. 3*E*) and 0.09 ± 0.01 for PKA alone (after PKD). This suggests that most of the Ca^2+^ desensitizing action of PKA-mediated bisphosphorylation of cTnI can be mimicked by PKD-mediated monophosphorylation alone. The maximum Ca^2+^-activated force (F_max_) generated by the trabeculae was unaffected by the incubation in PKD (Table S1). F_max_ showed a small but significant decrease with PKD+PKA incubation compared with that in dephosphorylated muscles, but this was not statistically significant compared with its corresponding time-matched control.

To check that the effect of PKD on Ca^2+^ sensitivity was mediated by phosphorylation at cTnI Ser-23/24, the experiments were repeated with λ-PP–treated skinned trabeculae from cTnI-Ala2 transgenic mice. Fig. 3*C* shows that Ca^2+^ sensitivity showed little or no change after incubation of these muscles with PKD or PKD+PKA. The *p*Ca_50_ fell by 0.10 ± 0.01 with PKD incubation, but this was not significantly different from the small decrease seen in the time-matched control (Fig. 3*D*). We conclude that the Ca^2+^-desensitizing action of PKD, like PKA, requires the presence of phosphorylatable Ser-23 or Ser-24 in cTnI.

In a previous study, we found that the monophosphorylation of cMyBP-C at Ser-302 by PKD could accelerate *k*_tr_ to the same extent as produced by trisphosphorylation of cMyBP-C (at Ser-273/282/302) by PKA (8). However, in that study, there was basal phosphorylation of all three cMyBP-C serines. We therefore repeated these experiments using λ-PP–treated skinned muscles. The rate of force redevelopment (*k*_tr_) varies with the degree of Ca^2+^ activation, *i.e.* with the relative force (19), so we measured *k*_tr_ over a range of forces and calculated the *k*_tr_ at a relative force of 50% of maximum (k_tr50_) by interpolation. The results are shown in Fig. S3 and Table S1. Although incubation in PKD alone tended to increase *k*_tr50_, this was not statistically significant (Fig. S3*D*). In contrast, subsequent incubation with PKA significantly increased relative *k*_tr50_ from 28.2% ± 3.6% (post-PKD) to 37.5% ± 2.1%. Similar results were found with experiments using cTnI-Ala2 mice (Fig. S3, *C* and *D*). There was no change in *k*_tr50_ in WT time-matched controls. To investigate why PKD failed to accelerate cross-bridge kinetics significantly in this study, phospho-specific antibodies were used to quantify the magnitude and pattern of cMyBP-C phosphorylation by PKD or PKA (Fig. S4). This confirmed our previous finding (8) that PKD phosphorylates cMyBP-C preferentially at Ser-302. However, in these λ-PP–treated myofibrils, the extent of phosphorylation of Ser-302 by PKD amounted to only about 40% of the phosphorylation of this same site by PKA after PKD. Thus, with these myofibrils, the PKD incubation conditions that produced full monophosphorylation of cTnI (Fig. 1) produced only partial phosphorylation of cMyBP-C Ser-302. Fig. S4 also illustrates the effectiveness of the initial λ-PP treatment in removing endogenous basal phosphorylation from these three serines of cMyBP-C.

### Protective effects of PKD and PKA on calpain-induced proteolysis

**Figure 4.**
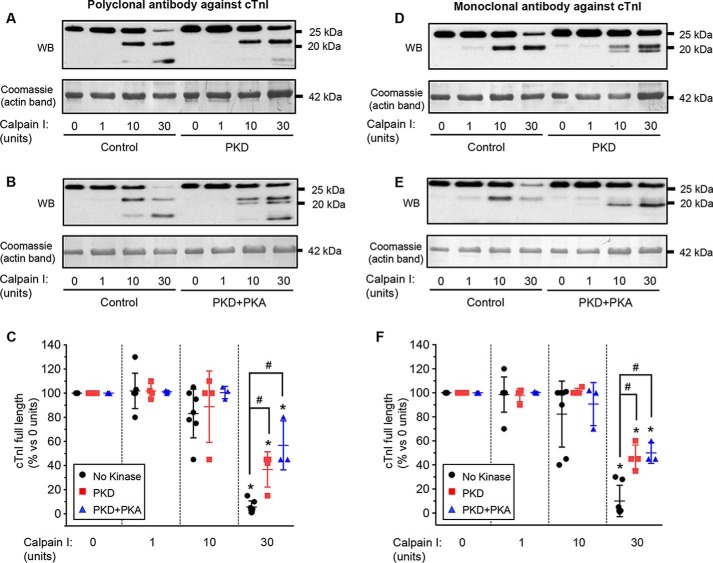
**Phosphorylation of sarcomeric cTnI by either PKD or PKA reduces its proteolysis by calpain.** *A*, λ-PP–treated isolated myofibrils were incubated with PKD alone and then treated with calpain-1 at the activities shown (as units per 100 μl). Shown is representative Western blotting (*WB*) using an antibody against full-length TnI and loading control (Coomassie stain). *B*, as *A*, except the myofibrils were incubated in PKD and then PKA before incubation in calpain. *C*, summary of calpain-dependent cTnI degradation after no kinase addition (*n* = 7), PKD alone (*n* = 4), or PKD+PKA (*n* = 3). *Symbols* show the data from individual experiments; *vertical bars* show the mean ± S.D. *D--F*, as *A–C* but using an antibody that recognizes a sequence in the C terminus of TnI. *Symbols* show the data from individual experiments; *vertical bars* show the mean ± S.D. (*n* = 6 for no kinase, *n* = 4 for PKD alone, *n* = 3 for PKD+PKA). *, *p* < 0.05 *versus* no calpain; #, *p* < 0.05 PKD or PKD+PKA *versus* no kinase addition.

Calpain has been implicated in the proteolysis of cTnI that can occur in cardiac disease. Phosphorylation of the isolated cardiac troponin complex by PKA slows the proteolysis of isolated cTnI by calpain (13). We investigated whether monophosphorylation by PKD or bisphosphorylation by PKA protects cTnI in the intact sarcomere from calpain-dependent degradation. Myofibrils were treated with λ-PP and then PKD alone or PKD followed by PKA. Calpain-1 was then added, and the pattern of cTnI degradation was measured by immunoblotting using a polyclonal antibody against the core sequences of troponin. Fig. 4, *A–C*, shows that incubation of the myofibrils in calpain-1 led to rapid proteolysis of cTnI, with near-complete loss of the full-length protein at the highest activity (30 units) of calpain-1 used. Calpain incubation produced two main degradation bands: the first band of a molecular mass of ∼22 kDa appeared with both 10 units and 30 units of calpain and sometimes was seen as a doublet; the second band was of lower molecular mass and became prominent only at the highest activity of calpain. Prior treatment of cTnI with PKD partially protected the full-length protein against subsequent calpain-induced proteolysis, particularly that seen at 30 units of calpain-1 (Fig. 4, *A* and *C*). Partial protection from proteolysis was also observed when cTnI was first treated with PKD+PKA (Fig. 4, *B* and *C*); in this case, about 50% of full-length cTnI was preserved after 30-min incubation in 30 units of calpain. The protective effects of PKD or PKD+PKA incubation were not significantly different (*p* > 0.05).

Previous reports have documented evidence for cTnI proteolysis within the C terminus or N terminus or both. To investigate whether the degradation products in our study were the results of N- or C-terminal cleavage of cTnI by calpain, we repeated our experiments using an mAb specific for residues 190–196 in the C terminus of cTnI (Fig. 4, *D–F*). The results confirmed that prior incubation in PKD or PKD+PKA partially protects cTnI from proteolysis (Fig. 4*F*). The C terminus antibody recognized the 22-kDa fragment(s) produced by calpain at 10 units or 30 units but not the lower-molecular-weight fragment seen with the polyclonal antibody at 30 units of calpain. This suggests that the first fragment retains the C terminus and thus is likely to be a product of N terminus degradation, whereas the second fragment lacks part or all of the C terminus amino acids 190–196, so is likely to be the result of proteolysis before or within this sequence. To explore the calpain proteolysis sites further, we used the pSer-23/24 antibody that recognizes pSer-23 and/or pSer-24 in the N terminus. This antibody recognizes full-length cTnI when the protein has been phosphorylated by incubation with PKD or PKD+PKA (Fig. 1, *C* and *D*). The results (Fig. S5) confirmed the gradual loss of the phosphorylated, full-length protein with calpain treatment, but the antibody failed to detect any of the degradation products, suggesting that both cTnI degradation products were missing the N-terminal sequence up to at least serines 23/24. Finally, we carried out the calpain experiments using myofibrils from cTnI-Ala2 mice (Fig. S6). With these myofibrils, the loss of full-length cTnI and the appearance of one or two degradation bands (depending on the antibody used) were unaffected by prior incubation with PKD+PKA; that is, the protective effect of phosphorylation was absent. Together, these data suggest that calpain leads to N-terminal and then C-terminal proteolysis of cTnI in the myofibrils and that PKD-induced monophosphorylation or PKA-induced bisphosphorylation of cTnI serines 23 and/or 24 can partially protect against this proteolysis.

Calpain is also known to cleave cMyBP-C in the N-terminal C0C2 domain, resulting in an N-terminal fragment (∼40 kDa). Pseudophosphorylation of cMyBP-C at serines 273, 282, and 302 in intact hearts protects against cleavage (20). Fig. S7 shows that 30 units of calpain produced near-complete loss of the full-length cMyBP-C and appearance of the low-molecular-weight fragment with a time course similar to the proteolysis of cTnI. Prior treatment of dephosphorylated cardiac myofibrils with PKD alone did not affect the proteolysis of cMyBP-C (Fig. S7*A*), but this result is ambiguous because PKD incubation only partially phosphorylated Ser-302 in cMyBP-C (Fig. S4). However, prior treatment with PKD+PKA provided a near-complete protective effect against calpain-induced cleavage (Fig. S7*B*), showing that PKA-mediated phosphorylation of cMyBP-C, like pseudophosphorylation, strongly protects this sarcomeric protein from calpain-mediated proteolysis.

### Phosphorylation of cTnI and cMyBP-C by PKD or PKA in intact myocytes

**Figure 5.**
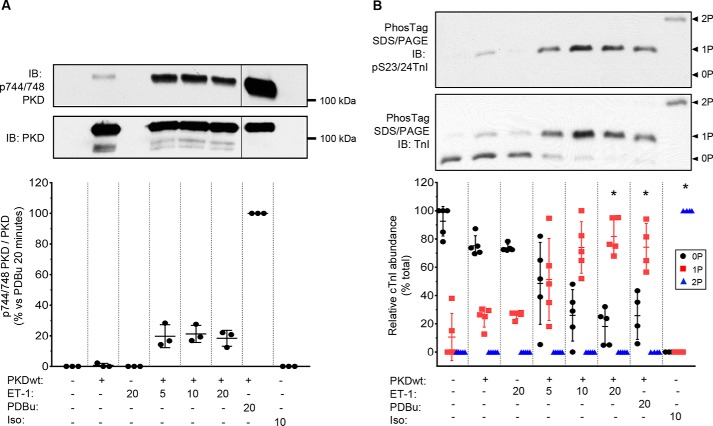
**PKD can monophosphorylate cTnI in rat adult cardiomyocytes.** Cardiomyocytes infected with adenovirus carrying PKDWT/EGFP (+) or control EGFP (−) were stimulated with ET-1 (100 nmol/liter for 5, 10, or 20 min), PDBu (200 nmol/liter, 20 min), or isoproterenol (*Iso*, 10 nmol/liter, 10 min). *A*, representative immunoblots (*IB*) of p744/748 PKD and total PKD (*top panel*; the *vertical line* shows where an empty lane has been removed) and relative levels of p744/748 PKD normalized to total PKD (*bottom panel*). *B*, representative Phos-tag gels of total TnI and phosphoserine 23/24 cTnI (*top panel*) and proportion of cTnI unphosphorylated, monophosphorylated, and bisphosphorylated cTnI after the various treatments. *Symbols* show the data from individual experiments; *vertical bars* show the mean ± S.D. (*n* = 3–5).

The experiments above were conducted using constitutively active, recombinant PKD as a tool to induce monophosphorylation. Although PKD activity is low in healthy adult cardiac tissue, the G_q_PCR/PKD pathway may become increasingly important in heart failure (21). To investigate whether full-length PKD can cause monophosphorylation in intact myocytes, we examined the phosphorylation of cTnI in adult rat ventricular myocytes (ARVMs) that had been transfected with adenovirus encoding full-length PKD1 48 h previously. In these myocytes, the PKC/PKD activators endothelin-1 and phorbol 12,13-dibutyrate produced modest and strong activation of PKD, respectively, as measured by phosphorylation of PKD Ser-744/748 (Fig. 5*A*). These agonists induced about 80% monophosphorylation of cTnI without any significant bisphosphorylation (Fig. 5*B*). In contrast, PKA activation by isoproterenol led to cTnI bisphosphorylation exclusively. Activation of PKD by PDBu or ET-1 also led to some phosphorylation of cMyBP-C at Ser-302, although the increase in phosphorylation level at this site was only 20–40% of that seen with activation of PKA (Fig. S8). These results showed that, in intact cardiomyocytes, interventions that activate PKD induce monophosphorylation of cTnI, as was the case with isolated myofibrils exposed to exogenous PKD.

## Discussion

Mammalian cardiac TnI has multiple potential phosphorylation sites that are targeted by a variety of kinases (1). Consequently, cTnI integrates the activity of several signaling pathways to regulate myofibril contractile function. That PKA phosphorylates cTnI at Ser-23 and Ser-24 is long-established, and it is widely held that phosphorylation of both sites is needed to produce the decrease in myofibrillar Ca^2+^ sensitivity. Here we provide evidence that this is not the case and that phosphorylation of just one site can regulate myofilament Ca^2+^ sensitivity. This has implications for the regulation of contraction under basal conditions and during inotropic stimulation. We also show that both mono- and bisphosphorylation of cTnI at Ser-23/24 can reduce its susceptibility to degradation by calpain, thereby providing a potential protective role in cardiac pathophysiology.

### Basal myofilament phosphorylation

A common problem with phosphorylation experiments with isolated cardiac myofibrils or skinned myocytes/muscles is the existence of a substantial and variable “basal” level of phosphorylation of sarcomeric proteins, particularly cTnI and cMyBP-C. With myofibrils from human tissue, the “healthy” heart samples usually derive from unused donor hearts from brain-dead individuals kept on inotropic support; consequently, cTnI Ser-23/24 phosphorylation in such samples can be high, *e.g.* about 1.3 mol/mol of cTnI (65% of maximum) (17, 22). However, even in diseased hearts, in which phosphorylation levels are lower, there can be substantial Ser-23/24 monophosphorylation (17, 23). With myofibrils from animal tissue, basal phosphorylation may be due to sympathetic stimulation during animal anesthesia, but it is seen even in tissue from β-blocker–treated animals (Fig. 1*A*), so it could be due to a basal activity of intracellular kinases. If functionally active, then this pre-existing phosphorylation would blunt the effects of exogenous kinases added experimentally. Prior attempts to fully dephosphorylate cTnI in the sarcomere using exogenously added phosphatases have been largely unsuccessful (24, 25). We report here that a pure, recombinant phosphatase like λ-PP can be used to produce full dephosphorylation of the Ser-23/24 sites on cTnI and of Ser-273/282/302 on cMyBP-C in cardiac myofibrils (Fig. 1 and Figure S4), provided that the incubation solution lacks EGTA (see supporting information). λ–PP requires Mn^2+^ for its activity (26), so the inhibitory effect of EGTA may result from chelation of this essential cofactor. The affinity of EGTA for Mn^2+^ is about 20 times greater than for Ca^2+^ (27), so even when millimolar total MnCl_2_ is added to relaxing solution, the free concentration of Mn^2+^ will be in the nanomolar range, well below the ∼5 μmol/liter *K_d_* of λ-PP for Mn^2+^ (26). Given that many other phosphatases require metal ions of Mn^2+^ or Zn^2+^ (which are also bound avidly by EGTA (27)) for catalytic activity, we advise that EGTA should be avoided in dephosphorylation solutions using these enzymes.

By putting the myofibrils into a “ground state” of zero phosphorylation in this way, we removed the confounding factor of pre-existing phosphorylation of the myofibrils. Subsequent incubation with PKD or PKA produced large decreases of myofibrillar Ca^2+^ sensitivity (total decrease by ∼0.32 *p*Ca units and 0.41, respectively; Fig. 3). These were much larger than in our previous study (decreases of 0.1 and 0.15) (8). It is probable that, in our previous study (and perhaps in similar studies by other groups), the basal phosphorylation, which was likely to be predominantly monophosphorylation (Fig. 1*A*), had depressed Ca^2+^ sensitivity substantially before any kinases were added; this would reduce the effects of kinases added subsequently. This illustrates the need for prior dephosphorylation if the full effects of phosphorylation on myofibrillar function are to be established.

### Monophosphorylation of cTnI decreases Ca^2+^ sensitivity

We showed previously that PKD decreases the Ca^2+^ sensitivity of skinned cardiac muscles, and we assumed that PKD, like PKA, acts via bisphosphorylation of Ser-23/24. This assumption was based on Western blotting with a pSer-23/24 antibody and on the absence of PKD's desensitizing effect in muscles from cTnI-Ala2 mice. For a more detailed and quantitative measurement of cTnI phosphorylation, in this work, we used Phos-tag gel analysis, which showed that PKD phosphorylates only one of the Ser-23/24 sites in mouse and human cardiac myofibrils (Figs. 1 and 2). Mass spectrometry analysis of cTnI confirmed this monophosphorylation. The ability of a cTnI pSer-23/24 antibody to detect PKD-mediated phosphorylation of cTnI in our previous study can be explained by the finding that this antibody recognizes not only the bisphosphorylated form of cTnI but also the monophosphorylated form (Fig. 2), supporting a previous observation (17). ARVM experiments (Fig. 5) showed that inotropic activation of full-length PKD in intact cells also leads to phosphorylation of only one site on cTnI.

Despite phosphorylating only one of the 23/24 serines, PKD nevertheless produced a large fall in Ca^2+^ sensitivity that was only slightly less than with cTnI bisphosphorylation with PKA (Fig. 3). Our finding that Ca^2+^ sensitivity is regulated by monophosphorylation disagrees with the orthodoxy that phosphorylation of both Ser-23 and Ser-24 is required to decrease Ca^2+^ sensitivity (*i.e.* that the 0P and 1P forms of cTnI are functionally equivalent and confer the same, higher Ca^2+^ sensitivity). This concept originates from a study by Potter and co-workers (5), who reconstituted a Tn complex containing cTnI Ser/Ala mutants into pig skinned muscles; they found that the PKA-induced fall of Ca^2+^ sensitivity was not observed when either Ser-23 or Ser-24 (or both) was mutated to nonphosphorylatable alanine. We cannot explain why our results differ from this, although we note that, as judged by the recovery of force, troponin reconstitution was only 40–70% (5). More recently, Wijnker *et al.* (6) used mutated human cTnI with phosphomimetic Ser→Asp substitutions to report that only the double phosphomimetic substitution of Ser-23/24 led to reduced Ca^2+^ sensitivity in human skinned myocytes; single-site substitutions had no effect. Although many studies have shown that substitution of serine with aspartate is functionally equivalent to serine phosphorylation, in fact, their side-chain structures are considerably different. The carbonyl group of aspartate has a net atomic weight of 44 and a charge of −1, whereas the phosphate group of phosphoserine has a net atomic weight of 95 and a charge at neutral pH of approximately −2 (28). Thus, from both size and charge considerations, a double Ser→Asp substitution at cTnI Ser-23/24 should be a better mimic of cTnI *mono*phosphorylation than of bisphosphorylation. Whether this explains the difference between the conclusions from our study and that of Wijnker *et al.* (6) is not clear. Species-specific differences cannot be ruled out. Another difference is that our model allowed us to compare the endogenous cTnI within the sarcomere when unphosphorylated, monophosphorylated, or bisphosphorylated, without the need for exchange of mutated cTnI.

Use of a pSer-24-specific antibody (17) helped to identify the major target site for PKD in cTnI (Fig. 2*C*). The similarity in the intensity of staining for the PKD-treated cTnI and the PKA-treated cTnI (in which the Ser-24 site was fully phosphorylated because there was only a 2P band), suggests that the phosphorylation in the PKD-treated human cTnI was predominantly (or entirely) at Ser-24. This agrees with our previous *in vitro* work on PKD substrate preference using cTnI Ser→Ala mutants reconstituted into a troponin complex with WT cTnT and cTnC (16). It may be that PKD phosphorylates Ser-23 significantly under other circumstances but not under the conditions and time scale of our experiments, which allowed us to probe the effects of monophosphorylation at the Ser-23/24 sites.

Most of the endogenous cTnI monophosphorylation in the native myofibrils was also at the PKD-targeted site (Ser-24) because PKD produced near-complete conversion of the 0P to the 1P form but almost no conversion of the 1P to the 2P form (Fig. 1*A* and Fig. S1). cTnI monophosphorylation has also been observed in human hearts (17, 23). In the intact, healthy adult cardiomyocyte, this basal phosphorylation is unlikely to be due to PKD, which is present only at a low level, but probably due to a residual activity of PKA or of other kinases known to phosphorylate cTnI at Ser-23/24 (29). Importantly, because this single site regulates Ca^2+^ sensitivity (as revealed by our PKD experiments), basal monophosphorylation in intact myocytes would provide a tonic regulation of basal Ca^2+^ sensitivity; it would also lessen the magnitude of any changes during subsequent inotropic activation, *e.g.* of PKA during β_1_-adrenoreceptor stimulation. We predict that the rate of Ca^2+^ dissociation from TnC, which determines Ca^2+^ sensitivity and influences myofilament relaxation rate, is also controlled by cTnI monophosphorylation. Finally, the increased Ca^2+^ sensitivity in skinned preparations from failing or hypertrophic cardiomyopathy hearts is presumed to be due to the decreased bisphosphorylation of cTnI (*e.g.* Ref. 30), but our study emphasizes the importance of changes in the monophosphorylated state of cTnI (17, 23). Fig. 5 shows that monophosphorylation can occur in cells with sufficiently active PKD; this may occur during α-adrenoreceptor stimulation in heart failure when the β-adrenoreceptor pathway is down-regulated (21, 31).

A limitation of our study is that the functional measurements were made with skinned trabeculae, but their low protein content precluded measurements of phosphorylation, which therefore were carried out with skinned myocytes/myofibrils. However, if myofibrillar Ca^2+^ sensitivity was controlled only by cTnI bisphosphorylation (5, 6), then, to account for our results, the same PKD incubation conditions that produced monophosphorylation in isolated myofibrils must have produced bisphosphorylation in the skinned muscles. We cannot see how this could occur. In fact, diffusional restrictions to enzyme movement might be predicted to slow the rate of cTnI phosphorylation in skinned muscles compared with isolated myofibrils.

We reported (8) that PKD increases *k*_tr_ by phosphorylating Ser-302 on cMyBP-C. However, here the trend for cross-bridge kinetics to increase was not statistically significant (Fig. S3). This is probably because only ∼40% phosphorylation of Ser-302 was observed, compared with ∼90% previously (8). The reduced phosphorylation of cMyBP-C by PKD may be due to loss of basal phosphorylation of Ser-282 during the λ-PP–mediated dephosphorylation because phosphorylation of Ser-282 appears to be necessary for the phosphorylation of Ser-302 *in vitro* (32). Thus, dephosphorylated cMyBP-C is a poorer PKD substrate than is dephosphorylated cTnI.

If the Ca^2+^ desensitizing action of cTnI bisphosphorylation is largely reproduced by monophosphorylation, why are both serines highly conserved (33)? One potential explanation is that bisphosphorylation may control a myofilament property not studied here, such as the sarcomere length dependence of Ca^2+^ sensitivity (Frank-Starling effect) (34). Another possibility is the monophosphorylated form of cTnI may be more susceptible than the bisphosphorylated form to the action of endogenous phosphatases; that is, bisphosphorylation may have a more prolonged effect.

### Protection from calpain-induced proteolysis

In intact hearts, proteolysis of cTnI occurs during ischemia/reperfusion, when C-terminal cleavage of cTnI forms a truncated cTnI_1–192_ that may contribute to stunning by decreasing the force-generating capacity of myofibrils (9, 10). Conversely, N-terminal cleavage of 26 or 27 amino acids from cTnI was observed in chronic simulated microgravity (11), heart failure (12), and even in normal hearts (12) and mimicked Ser-23/24 phosphorylation by reducing myofibrillar Ca^2+^ sensitivity and accelerating muscle relaxation. Ca^2+^-activated calpain may be responsible for the C-terminal (9) or the N-terminal (12) cleavage. We found that calpain degrades sarcomeric cTnI, forming two major TnI degradation products. The pattern and time course of cTnI degradation (Fig. 4 and Figs. S4–S6) suggested that the initial product resulted from cTnI N-terminal cleavage at a site after Ser-23; this was then further degraded within the C terminus to form the second product. This sequence is contrary to that found by McDonough *et al.* (9), who reported that I/R in rat hearts produced initial C-terminal cleavage to form 22-kDa cTnI_1–193_, with additional N-terminal cleavage to form fragments cTnI_63–193_ and cTnI_73–193_ under more severe conditions. However, during I/R, other proteases may be activated, and the low pH could alter the pattern of calpain-induced degradation. The initial N-terminal degradation products we observed are similar to those (21 kDa and 22 kDa) found in intact hearts during simulated microgravity (11) and heart failure (12). An initial N-terminal cleavage was also found with calpain-induced proteolysis of recombinant cTnI (35). The two main N-terminal cleavage products in the intact hearts resulted from the loss of amino acids 1–26 and 1–27, but we have no direct information regarding the exact sites of cleavage in our study.

Calpain-induced proteolysis in the isolated troponin complex is partly inhibited after phosphorylation of cTnI by PKA (13). Here we confirm that bisphosphorylation of cTnI in intact sarcomeres has a substantial, although incomplete, protective effect (Fig. 4). A similar protective effect was also conferred by monophosphorylation with PKD alone. If the first cleavage is at N-terminal residues 26 or 27, then phosphorylation at serines 23 and/or 24 might interfere sterically with the nearby binding of calpain. Alternatively, because phosphorylation at one or both serines alters cTnI structure sufficiently to produce a large fall of Ca^2+^ sensitivity, the tertiary structure and flexibility of the regulatory N-terminal sequence will be changed, and this could inhibit binding of calpain.

Like cTnI, cMyBP-C is degraded during myocardial ischemia, although in this case, proteolysis occurs in the N terminus, forming a 40-kDa C0C1f fragment that may act as a poison peptide (20). cMyBP-C is a substrate for calpain, and pseudophosphorylation of the three serines within this sequence (273, 282, and 302) makes the protein less susceptible to proteolysis (14, 15). We found that the native cMyBP-C protein within the sarcomere is degraded by calpain with a time course similar to that for proteolysis of cTnI (Fig. S7). Trisphosphorylation of cMyBP-C with PKA, like pseudophosphorylation, provided complete protection against this proteolysis, at least under the conditions of our experiment. We were unable to establish whether monophosphorylation of cMyBP-C (at Ser-302) by PKD also has a protective effect because PKD produced only a low level of cMyBP-C Ser-302 phosphorylation under the incubation conditions used.

In summary, our results demonstrate that monophosphorylation of cTnI, primarily at Ser-24, affects both myofibrillar Ca^2+^ sensitivity and the susceptibility of cTnI to calpain-induced proteolysis. These effects are more similar functionally to those conferred by cTnI in its bisphosphorylated form, not in its unphosphorylated form.

## Experimental procedures

An expanded Methods section is provided in the supporting information.

### Ethics statement

Experiments using mouse trabeculae and rat cardiomyocytes were performed in accordance with the Guidance on the Operation of Animals (Scientific Procedures) Act, 1986 (UK) and the Directive of the European Parliament (2010/63/EU) and received approval from the King's College London Ethics Review Board. Human samples were obtained after informed consent and with approval of the Human Research Ethics Committee of the University of Sydney (approval 2012/2814). This investigation conformed with the principles in the Declaration of Helsinki (1997).

### Assessment of myofibril contractile function in skinned trabeculae

The methods for preparing mouse skinned trabeculae and for measuring their Ca^2+^ sensitivity and cross-bridge cycling kinetics have been described previously (8). In brief, thin trabeculae were prepared from the right ventricles of WT mice or cTnI-Ala2 transgenic mice (mice that express cTnI in which Ser-23 and Ser-24 are replaced by two nonphosphorylatable Ala residues on a cTnI-null background (8)) and were permeabilized with Triton X-100. The skinned trabeculae were bathed in relaxing solution (free [Ca^2+^], ∼1 nmol/liter; *p*Ca, ∼9) and clamped to a force transducer and high-speed length controller. Force and cross-bridge cycling kinetics were measured at 18 °C in a series of calcium/EGTA activating solutions containing 0.2–30 μmol/liter free Ca^2+^ (*p*Ca 6.7–4.5). Cross-bridge cycle kinetics were assessed by performing a rapid release–restretch maneuver to forcibly detach the cross-bridges; the *k*_tr_ when cross-bridges reattach and generate force after the restretch was used as an index of cross-bridge kinetics (8, 36, 37).

### Initial dephosphorylation of myofibrils

Although the mice were treated with a β-adrenoreceptor antagonist before euthanasia (see supporting information), the detergent-treated muscles or isolated myofibrils nevertheless exhibited substantial endogenous phosphorylation of cTnI (Fig. 1*A*). To remove this endogenous phosphorylation, we first incubated the skinned trabeculae in λ-PP. However, in pilot experiments we found that λ-PP was inhibited in relaxing solution, probably because of binding of its Mn^2+^ cofactor by EGTA (see “Discussion”). On the other hand, EGTA is normally required in relaxing solution to avoid a large and potentially damaging Ca^2+^-activated contracture caused by contaminant Ca^2+^. We therefore devised a novel dephosphorylation procedure in which the trabeculae were first put into a low-force rigor contracture by removing ATP in the presence of EGTA, and then EGTA was removed and λ-PP applied. Upon return to relaxing solution, the muscle and its sarcomeres showed no visible damage. This sequence of solutions, but performed instead with skinned myocyte fragments and myofibrils (see supporting information), led to almost complete dephosphorylation of cTnI (Fig. 1, *B* and *C*) and cMyBP-C (Fig. S4). The same protocol was used to prepare dephosphorylated myofibrils from left ventricular samples from human (unused donor) hearts.

### Contractile effects of PKD- and PKA-induced phosphorylation

The overall experimental protocol consisted of first measuring the Ca^2+^ sensitivity of force and *k*_tr_ in the trabeculae using the standard relaxing and activating solutions after incubation in λ-PP, then again after incubation in constitutively active PKD, and finally (for a subset of muscles) after incubation in PKA catalytic subunit. The phosphatase inhibitor calyculin A was included in all solutions following λ-PP incubation. With this protocol, each trabecula served as its own control, allowing paired comparison of pre- and post-kinase data. Time-matched controls were included, in which λ-PP-treated muscles were subjected to the identical incubation protocols except that PKD and PKA were omitted.

### Assessment of myofibril protein phosphorylation

The trabeculae contained insufficient tissue to allow measurement of their myofibril phosphorylation, so instead we prepared suspensions of skinned myocyte fragments and myofibrils (herein collectively referred to as myofibrils) by homogenization of ventricular tissue. Myofibrils were treated with the same dephosphorylation/phosphorylation protocols and solutions as used for the trabeculae. The extent and pattern of phosphorylation of cTnI and cMyBP-C in the myofibrils was measured by immunoblot analysis after either standard SDS-PAGE or Phos-tag^TM^ phosphate affinity SDS-PAGE. For one set of PKD-treated myofibrils, we assessed the phosphorylation of cTnI using high-resolution MS (38).

### PKD- or PKA-induced phosphorylation in cultured ARVMs overexpressing PKD

AVRMs were isolated and infected with a PKD1WT-EGFP adenovirus or EGFP adenovirus control (39). After 48 h in culture, ARVMs were bathed in a HEPES–Krebs solution and stimulated with isoproterenol (10 nmol/liter), endothelin-1 (ET-1, 100 nmol/liter), or phorbol 12,13-dibutyrate (PDBu, 200 nmol/liter). Myofibril phosphorylation was then determined.

### Source of chemicals

#### 

##### Primary antibodies

Cell Signaling Technology supplied the phosphospecific antibody against pSer-23/24 cTnI (4004) and the rabbit anti-TnI polyclonal antibody (4002) that was used to label total TnI. The phosphospecific antibody against pSer-24 of human cTnI was a kind gift from Steven Marston. A mouse anti-cTnI mAb, with an epitope of residues 190–196 of cTnI, was from Hytest (MF4). Phosphospecific antibodies against pSer-273, pSer-282, and pSer-302 of cMyBP-C (40) were kind gifts from Sakthivel Sadayappan and Jeffrey Robbins. Primary antibodies against PKD phosphoserine 744/748 (2054) and total PKD (C20) were obtained from Cell Signaling Technology and Santa Cruz Biotechnology, respectively.

##### Adenovirus

A recombinant adenovirus (AdV:PKDWT/EGFP) encoding full-length WT mouse PKD1 (PKDWT) and enhanced GFP (EGFP) downstream of separate cytomegalovirus promoters was as described previously (39).

##### Enzymes

PKA catalytic subunit (539576, from bovine heart) was from Calbiochem/Merck-Millipore, and λ phosphatase was from New England Biolabs (P0753S, 20,000 units/μg). PKD was a recombinant PKD1 catalytic domain expressed in Sf21 insect cells; this is constitutively active in the absence of the autoinhibitory N-terminal domain (16). Calpain (1000 units/mg, from human erythrocytes) and endothelin-1 were from Calbiochem. Other reagents are detailed in the supporting data.

### Statistics

Graphical statistics are shown as individual symbols and mean ± S.D.; some numerical data are listed as mean ± S.E. Statistical comparisons were by paired or unpaired Student's *t* test, as appropriate, when comparing data between two groups or by analysis of variance followed by the Bonferroni test when comparing data between multiple groups. *p* < 0.05 was considered statistically significant.

## Author contributions

A. M.-G., M. A., and J. C. K. conceptualization; A. M.-G., Y. S., C. G. d. R., S. A.-G., W. C., Y. G., M. A., and J. C. K. data curation; A. M.-G., Y. S., C. G. d. R., S. A.-G., and J. C. K. formal analysis; A. M.-G., Y. S., C. G. d. R., S. A.-G., W. C., Y. G., M. A., and J. C. K. validation; A. M.-G., B. J. B., H. E. S., Y. S., C. G. d. R., S. A.-G., W. C., and M. A. investigation; A. M.-G., Y. S., C. G. d. R., S. A.-G., W. C., Y. G., and J. C. K. visualization; A. M.-G., B. J. B., H. E. S., Y. S., C. G. d. R., S. A.-G., W. C., M. A., and J. C. K. methodology; A. M.-G., B. J. B., H. E. S., Y. S., C. G. d. R., S. A.-G., Y. G., M. A., and J. C. K. writing-review and editing; B. J. B., H. E. S., Y. S., C. G. d. R., W. C., Y. G., M. A., and J. C. K. resources; B. J. B., C. G. d. R., Y. G., M. A., and J. C. K. funding acquisition; B. J. B., Y. G., M. A., and J. C. K. project administration; W. C., Y. G., M. A., and J. C. K. software; Y. G., M. A., and J. C. K. supervision; J. C. K. writing-original draft.

## Supplementary Material

Supporting Information

## Footnotes

5 A. Martin-Garrido, B. J. Biesiadecki, H. E. Salhi, Y. Shaifta, C. G. dos Remedios, S. Ayaz-Guner, W. Cai, Y. Ge, M. Avkiran, and J. C. Kentish, unpublished results.

## Footnotes

4 The abbreviations used are:

PKA: protein kinase A
PKD: protein kinase D
cTnI: cardiac troponin I
cMyBP-C: cardiac myosin–binding protein C
I/R: ischemia/reperfusion
λ-PP: lambda phosphatase
*p*Ca: −log of free Ca^2+^ concentration
*p*Ca_50_: *p*Ca for 50% activation of force
*k*_tr_: rate constant of tension (force) redevelopment
*k*_tr50_: *k*_tr_ at 50% activation of force
ARVM: adult rat ventricular myocyte
PDBu: phorbol 12,13-dibutyrate
EGFP: enhanced GFP.
