# Supplementary material for: Monophosphorylation of cardiac troponin-I at Ser-23/24 is sufficient to regulate cardiac myofibrillar Ca2+ sensitivity and calpain-induced proteolysis
Source: J Biol Chem. 2018 Apr 18;293(22):8588–99. doi: 10.1074/jbc.RA117.001292 (PMC5986213; doi:10.1074/jbc.RA117.001292)
Supplement: Supporting Information [file supp_293_22_8588__index.html]

Monophosphorylation of cardiac troponin-I at Ser23/24 is sufficient to regulate cardiac myofibrillar Ca2+ sensitivity and calpain-induced proteolysis — Effects of cardiac TnI monophosphorylation at Ser23/24 — Monophosphorylation of cardiac troponin-I at Ser-23/24 is sufficient to regulate cardiac myofibrillar Ca2+ sensitivity and calpain-induced proteolysis — Effects of cardiac TnI monophosphorylation at Ser-23/24 — Supporting Information 

# Monophosphorylation of cardiac troponin-I at Ser-23/24 is sufficient to regulate cardiac myofibrillar Ca2+ sensitivity and calpain-induced proteolysis

## Supporting Information

- JBC-2107-001292-R2 Supplementary Material.pdf - Supporting material (methods and results).
