# Supplementary material for: Monophosphorylation of cardiac troponin-I at Ser-23/24 is sufficient to regulate cardiac myofibrillar Ca2+ sensitivity and calpain-induced proteolysis
Source: J Biol Chem. 2018 Apr 18;293(22):8588–99. doi: 10.1074/jbc.RA117.001292 (PMC5986213; doi:10.1074/jbc.RA117.001292)
Supplement: Supporting Information [file supp_RA117.001292_134423_2_supp_116270_p6yxwg.pdf]

SUPPLEMENTARY MATERIAL

Monophosphorylation of Cardiac Troponin-I at Ser23/24 is Sufficient to Regulate Cardiac Myofibrillar Ca^{2+} Sensitivity and Calpain-Induced Proteolysis

Abel Martin-Garrido, Brandon J. Biesiadecki, Hussam E Salhi, Yasin Shaifta, Cristobal dos Remedios, Serife Ayaz-Guner, Wenxuan Cai, Ying Ge, Metin Avkiran, & Jonathan C Kentish

Contents

Page

1. Detailed methods S-1
2. Supplemental figures S-6
3. Supplemental table S-14
4. References S-15

1. Detailed Methods

Preparation of skinned trabeculae

The methods for preparing mouse skinned trabeculae and for measuring their Ca^{2+} sensitivity and crossbridge cycling kinetics have been described in detail previously (1);new or altered methods are described below. All procedures were carried out in accordance with institutional and national guidance on the humane care of animals. In brief, wild-type (WT) mice or cTnI-Ala2 mice transgenic (mice that express cTnI in which Ser23 and Ser24 are replaced by two non-phosphorylatable Ala residues on a cTnI-null background (1)) were given the β-adrenoceptor antagonist, propranolol (Sigma, 0.5 g/L), in the drinking water for 3 days before sacrifice, in an attempt to reduce basal phosphorylation of myofibrils, e.g. (2-4). This dose and duration of treatment was based on our earlier work (1). After heparinization and euthanization of the mice (500 IU heparin, 33mg/kg sodium pentobarbital), their hearts were excised and rinsed thoroughly in ice-cold HEPES-Krebs solution (containing, in mmol/L: NaCl 130, $MgCl_2$ 4.5, NaH_2PO_4 0.4, $CaCl_2$ 0.75, HEPES [1] 4.2, taurine 20, creatine 10 and glucose 10). The atria were removed and the ventricles were blotted gently on tissue paper before being frozen directly in liquid nitrogen. (Blotting was found to be essential, because any liquid remaining in the ventricles when they were frozen often led to fracture of the ventricular wall, including the trabeculae). The ventricles were then transferred to pre-cooled cryovials for storage in liquid nitrogen.

---

[1] Abbreviations used are: HEPES, N-[2-hydroxyethyl]piperazine-N'-[2-aminoethanesulfonic acid]; BES, N,N-Bis(2-hydroxyethyl)-2-aminoethanesulfonic acid; DTT, dithiothreitol; E64, trans-Epoxysuccinyl-L-leucylamido(4-guanidino)butane; AEBSF, 4-(2-Aminoethyl)benzenesulfonyl fluoride hydrochloride; PKA, protein kinase A; PKD, protein kinase D; cTnI, cardiac troponin I; cMyBP-C, cardiac myosin-binding protein C; RSK, p90 ribosomal S6 kinase; λ–PP, lambda phosphatase; pCa, -log of free Ca^{2+} concentration; EGFP, Enhanced green fluorescent protein.

Assessment of myofibril contractile function

On the day of the experiment, the ventricles were thawed in relaxing solution containing (in mmol/L): 100 BES, 50 K propionate, 10 EGTA, 10 Na_2 phosphocreatine, 5 $MgATP^{2-}$, 1 Mg^{2+}, 1 dithiothreitol, and 0.001 leupeptin, 0.001 E64, and 0.25 AEBSF; pH 7.1; ~1 nmol/L Ca^{2+} (pCa 9); 4°C. Ca/EGTA and other solutions were prepared as described in detail previously (5). Thin trabeculae (diameter usually < 100 µm – see table for details) were dissected from the right ventricle, “skinned” with 1% Triton X-100 in relaxing solution for 30 minutes, and clamped to a 403A force transducer and 308B high-speed length controller (both from Aurora Scientific). The muscle bath was mounted on a Nikon inverted microscope. Sarcomere length (SL) was set to 2.2 µm in relaxing solution (pH 7.1 at 18°C), using the muscle’s video image and IonOptix software. Force and crossbridge cycling kinetics were measured in a series of Ca/EGTA activating solutions containing 0.2 – 30 µmol/L free Ca^{2+} (pCa range 6.7–4.5), with relaxation solution applied between each activation. Ca^{2+}-activated force was calculated as the steady-state force achieved during the contraction minus the passive force in relaxing solution. Cross-bridge cycle kinetics at each Ca^{2+} concentration were assessed by performing a release-restretch maneuver during steady Ca^{2+} activation: the muscle was rapidly slackened by 20% of its length for 20 ms and then restretched to its initial length. This maneuver forcibly detaches the cross-bridges. The rate of force redevelopment as crossbridges re-attach and generate force after the restretch (k_{tr}) was measured from a single-exponential fit to the force data and was used as an index of cross-bridge kinetics (1),(6,7).

To determine the Ca^{2+}-sensitivity of force, submaximal Ca^{2+}-activated force at each free Ca^{2+} concentration was expressed as a fraction of the maximal Ca^{2+}-activated force at 30 µmol/L Ca^{2+}(pCa 4.5). The force-Ca^{2+} curves were fitted by the following modified Hill equation using Origin software (OriginLab Corp.), to determine the Hill coefficient (nH), free Ca^{2+} concentration at 50% of maximum force (EC50) and pCa50:

Relative force = Maximum force x $[Ca^{2+}]^{nH} / (1 + [Ca^{2+}]^{nH})$.

Force values were normalized to cross-sectional area, by measuring the width of the trabecula and assuming a cylindrical cross section. The k_{tr} value measured at each submaximal free calcium concentration was expressed as a fraction of the maximal k_{tr} value measured in maximum activating solution (30 µmol/L Ca^{2+}). Relative k_{tr} was then plotted against relative force to determine the effects of kinase treatment. Relative k_{tr}-relative force data were fitted to a polynomial curve to determine the relative k_{tr} at 50% of maximum force.

Initial dephosphorylation of myofibrils

To remove endogenous phosphorylation, we incubated the skinned trabeculae in lambda protein phosphatase (λ–PP) in an EGTA-free solution. The skinned trabecula was first transferred from relaxing solution into an “EGTA-rigor” solution (composition in mmol/L: 50 BES, 160 KCl, 2 DTT, 10 EGTA, 0.001 leupeptin, 0.001 E64, 0.25 AEBSF; pH 7.1 at 22°C) for 2 minutes, during which time a small rigor contracture developed (“low-rigor state” (8)). The trabecula was then washed three times in “phosphatase buffer” (composition as for EGTA-rigor solution but without EGTA and with 3 mmol/L $MnCl_2$ added) before being incubated in phosphatase buffer containing λ–PP (1000 U/mL) for 45 minutes at 22°C. During this incubation, the small rigor force stayed constant or slowly declined (not shown). On return to relaxing solution, any residual rigor contraction relaxed fully. The muscle and its sarcomeres showed no visible damage. The phosphatase inhibitor calyculin A (50 nmol/L)

was added to all solutions following λ–PP incubation. The same sequence of solutions, but using skinned myocyte fragments and myofibrils (see below), led to almost complete dephosphorylation of the "PKA sites" of cTnI (Fig. 1) and cMyBP-C (Supplementary Fig. S4).

Contractile effects of phosphorylation with PKD and PKA

The overall experimental protocol consisted of measuring the Ca^{2+} sensitivity of force and crossbridge kinetics (k_{tr}) in the trabecula using the standard relaxing and activating solutions (above) after incubation of the trabecula in λ–PP, then again after incubation in constitutively-active PKD (1 hour at 18°C), and finally after incubation in PKA catalytic subunit (5 U/μL; 1 hour at 18°C). The PKD used had a similar activity to that of PKA (1). With this protocol, each trabecula served as its own control, allowing paired comparison of pre- and post-kinase data. To check whether there were phosphorylation-independent changes in myofibril contractile properties over the course of an experiment, we included time-matched controls in which λ–PP-treated muscles were subject to the identical incubation protocols except that PKD and PKA were omitted. Data were discarded if maximum force declined by greater than 15% between consecutive kinase incubations or time-matched incubations, or if the SL did not recover to ~2.2 μm in relaxing solution after maximum activation (pCa 4.5).

PKD- or PKA-induced phosphorylation in cultured adult rat ventricle myocytes (ARVM) infected with PKD

Isolation and culture of AVRM and infection with a PKD1/EGFP adenovirus were as described previously (9). The recombinant adenovirus (AdV:PKDwt/EGFP) encoded full-length wild-type mouse PKD1 (PKDwt) and enhanced green fluorescent protein (EGFP), downstream of separate CMV promoters. Isolated ARVM were plated in 6-well plates coated with laminin (Sigma). After 2 hours the number of myocytes per well was counted and myocytes were infected to a MOI of 100 with the PKDwt/EGFP adenovirus or empty vector (control). After 48 hours in culture in modified M199 medium, ARVM were transferred to a HEPES-Krebs solution (composition as above) and were stimulated with one of the following: isoproterenol (10 nmol/L) for 5 min; endothelin-1 (ET-1; 100 nmol/L) for 5, 10 or 20 min; phorbol 12,13-dibutyrate (PDBu; 200 nmol/L) for 20 min at 37°C. In control experiments, cells received vehicle for 20 min. The reactions were stopped by aspirating the medium and adding Laemmli buffer (2x concentrated) directly to the wells and boiling (98°C) for 7 min. Myofibril phosphorylation was determined as described below.

Assay for calpain-mediated degradation of cTnI or cMyBP-C

Using the protocols described above, myofibrils were incubated with λ–PP and then PKD, with a subset treated subsequently with PKA. Time-matched controls (no kinase) were included. After the final incubation, myofibrils were centrifuged (4400 rpm for 5 min) and the pellet washed twice with Ca/EGTA activating solution of 2.5 μmol/L free Ca^{2+} (pCa 5.61) without protease inhibitors. The pellet was resuspended in the same solution (100 μL per reaction). Different amounts of calpain 1 (1 – 30 U added per reaction) were added to the myofibril suspension for 15 minutes at 22°C before the reaction was stopped by adding concentrated (3x) Laemmli buffer and boiling (98°C) for 7 min. Gels of the myofibrils were run and stained with Coomassie or with antibodies specific for TnI or cMyBP-C. The optical density of the full-length protein band was quantified by scanning autoradiograms with a calibrated densitometer (GS-800), using Quantity One® 1-D analysis software (Bio-Rad).

Preparation of skinned myocyte fragments and myofibrils

Skinned myocyte fragments and myofibrils (hereafter termed myofibrils) were treated with the same dephosphorylation/phosphorylation protocols and with the same solutions as were used for the skinned trabeculae. Some myofibril suspensions were prepared from the ventricular tissue that remained after the dissection of trabeculae; this ventricular tissue was snap-frozen in liquid nitrogen. Other myofibril suspensions were prepared directly from frozen ventricles (described above) that were not used for trabecula experiments. (The results were the same in each case). The frozen ventricular tissue was crushed with a stainless-steel mortar and pestle at liquid nitrogen temperature and then homogenized in relaxing solution to which was added 1% Triton X-100. In initial experiments we used a glass hand-homogeniser to produce myofibrils and myocyte fragments, but in later experiments we homogenized using a Polytron (PT 10-35GT; 3 times for 20s each at 13000 rpm), because this gave a more consistent loading of the gels. The phosphorylation results were the same for both methods. After 30 min in the Triton X-100 solution (on ice), the homogenate was centrifuged at 4400 rpm at 4°C for 10 minutes, then the pellet was washed and resuspended in EGTA-rigor solution. After 2 minutes, the myofibrils were centrifuged (4400 rpm for 10 minutes), washed twice in phosphatase buffer and resuspended in phosphatase buffer containing λ–PP (1000 units per ml). After 45 minutes of incubation at 22°C, myofibrils were re-suspended in relaxing solution, with this step repeated a further two times to remove any traces of λ-PP. The myofibrils were then incubated for 60 min at 18°C in relaxing solution containing the phosphatase inhibitor calyculin (50 nmol/L) and constitutively-active PKD (1) or no kinase (time-matched control). In some experiments, isolated myofibrils were further incubated with calyculin-containing relaxing solution containing the catalytic subunit of PKA (5 U/µL) or no kinase (time-matched) for 60 min at 18°C. Subsequently, myofibrils were washed with PBS twice (to remove EGTA that can interfere with PhosTag gels), re-suspended in Laemmli sample buffer and heated to 98°C for 7 minutes, for subsequent immunoblot analysis.

Human cardiac myofibrils were prepared from left ventricular samples of three unused human donor hearts that had been collected by the Sydney Heart Bank with full ethical permission. These samples had been snap-frozen and stored in liquid nitrogen. On the day of an experiment, a portion of a frozen tissue sample was cut into small fragments using a razor blade and the fragments were homogenised using the Polytron technique above. Human myofibrils were then prepared as described above for the mouse myofibrils.

The extent and pattern of phosphorylation of cTnI and MyBP-C in the mouse or human myofibrils were measured by immunoblot analysis after either standard SDS-PAGE or PhosTag phosphate affinity SDS-PAGE.

SDS-PAGE and immunoblot analysis

In brief, myofibril preparations in Laemmli sample buffer were separated by 7.5%, 12% or 15% SDS-PAGE, transferred to polyvinylidene difluoride (PVDF) membranes and subjected to immunoblotting (see below for details of the primary antibodies). These antibodies were detected by anti-rabbit or anti-mouse secondary antibody linked to horseradish peroxidase and enhanced chemiluminescence (Cell Signaling). Phosphorylation status of specific protein bands was quantified by scanning autoradiograms with a calibrated densitometer (GS-800), using Quantity One® 1-D analysis software (Bio-Rad).

Phos-tag phosphate affinity SDS-PAGE

Phos-tag phosphate affinity SDS/PAGE was performed essentially as described previously.(1) Protein samples were separated by electrophoresis at 80 volts on 8% SDS-PAGE gels containing Phos-tag reagent (50 µmol/L) and $MnCl_2$ (100 µmol/L). Prior to protein transfer, Mn^{2+} ions were chelated from the gel by incubation in transfer buffer containing 2 mmol/L EDTA for 30 min on an orbital shaker, before incubation of the gel in transfer buffer alone to remove EDTA for a further 30 min. Proteins were transferred to PVDF membranes and detected by standard immunoblot techniques, as described above.

Top-down high-resolution mass spectrometry analyses

Myofilament proteins were separated with a home-packed PLRP column (PLRP-S, 200 mm x 500 µm, 10 µm, 1000 Å; Varian, Lake Forest, CA, USA) using the Dionex U3000 LC system (Thermo Scientific, Bremen, Germany). The separation was performed with a gradient going from 20% solvent B to 90% solvent B (solvent A: 0.10% formic acid in water; solvent B: 0.10% formic acid in a 50:50 mixture of acetonitrile and ethanol) over 40 min at a flow rate of 12.5 µL/min. The proteins eluted were analyzed on-line with an Orbitrap Fusion Lumos Tribrid mass spectrometer (Thermo Scientific) operated in intact protein mode with a resolution setting of 240,000 (at 200 m/z). The MS data were analysed using the vendor-specific software Xcalibur 3.1 (Thermo Scientific) and the MASH Suite Pro software developed in-house (10).

Source of chemicals

Primary antibodies

Cell Signaling Technology supplied the phosphospecific antibody against pSer23/24 cTnI (#4004) and the rabbit anti-TnI polyclonal antibody (#4002) that was used to label total TnI. The phosphospecific antibody against pSer24 of human cTnI was a kind gift from Steven Marston. A mouse anti-cTnI monoclonal antibody, with an epitope of residues 190-196 of cTnI, was from Hytest (MF4). Phosphospecific antibodies against p273, p282, and pSer302 of cMyBP-C (11), and against cMyBP-C, were a kind gift from Sakthivel Sadayappan and Jeffrey Robbins. Primary antibodies against PKD phosphoserine 744/748 (#2054) and total PKD (C20) were obtained from Cell Signaling Technology and Santa Cruz, respectively.

Adenovirus

A recombinant adenovirus (AdV:PKDwt/EGFP) encoding full-length wild-type mouse PKD1 (PKDwt) and enhanced green fluorescent protein (EGFP) downstream of separate CMV promoters was as described previously (9).

Reagents

PKA catalytic subunit (#539576, from bovine heart) was from Calbiochem/Merck-Millipore and lambda phosphatase from New England Biolabs (#P0753S, 20000 U/µg). A constitutively-active, recombinant form of PKD1 was as used previously (12). Calpain (1000 U/mg, from human erythrocytes) and endothelin-1 were from Calbiochem. Phos-tag™ acrylamide (AAL-107) was from Alpha Laboratories Ltd. Isoproterenol was from Sigma-Aldrich. Other chemicals were from Calbiochem, Sigma-Aldrich, Roche Diagnostics, Invitrogen or VWR International.

2. Supplemental Figures

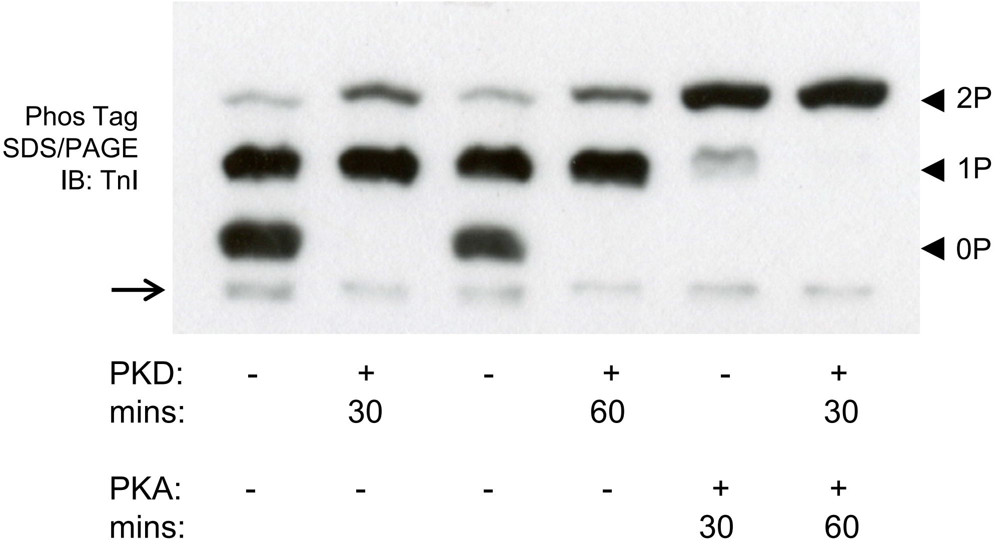

Figure S1: Phosphorylation of cTnI in myofibrils by PKD alone or PKD followed by PKA. Isolated myofibrils (without λ-PP treatment) were incubated in PKD and/or PKA for 30 or 60 mins, separated in SDS-PAGE with Phos-tag reagent, and blotted using a polyclonal antibody against TnI. The gel was over-exposed to show up any weak bands (note that the strong bands are saturated). 0P, 1P and 2P refer to bands we assigned as the unphosphorylated, mono-phosphorylated and bis-phosphorylated forms of intact cTnI, respectively. In this over-exposed gel a fourth band (left-hand arrow) could be seen, but this was of lower MW and was unaffected by incubation in PKD or PKA; this band may be due to cTnI that has suffered N-terminal degradation or it could be a residual amount of the fetal form of TnI (slow skeletal TnI), which lacks the phosphorylatable N-terminal sequence. There was no evidence for a cTnI band of higher MW than the 2P band after incubation in PKD ± PKA.

The disappearance of the 0P band after incubation in PKD and the only slight increase in the 2P band suggests that nearly all of the monophosphorylation in cTnI that was present in the native myofibrils before PKD treatment existed at the same serine residue that was targeted by PKD.

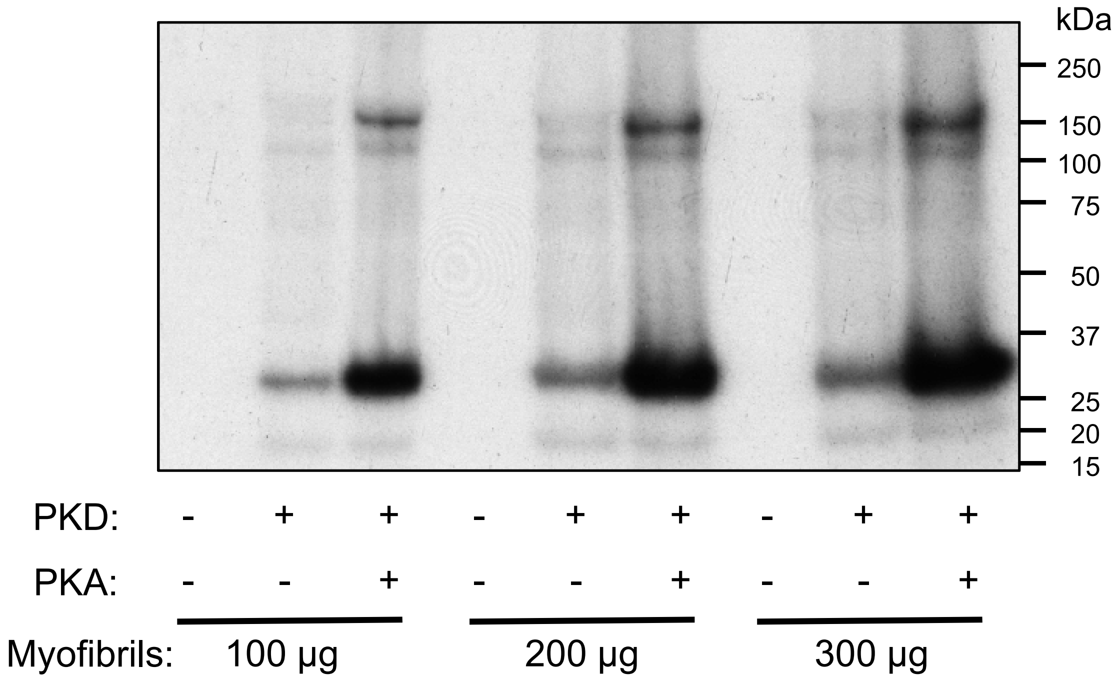

Figure S2: Proteins phosphorylated by PKD or PKD+PKA in isolated myofibrils. Myofibrils were incubated with λ-PP and then incubated in PKD alone (1 hour, 18°C), or PKD then PKA (each 1 hour, 18°C), in relaxing solution containing 32P-ATP (10 µCi per 200 µL). Different quantities (100 – 300 µg) of myofibrils were separated with SDS-PAGE and exposed to X-ray film.

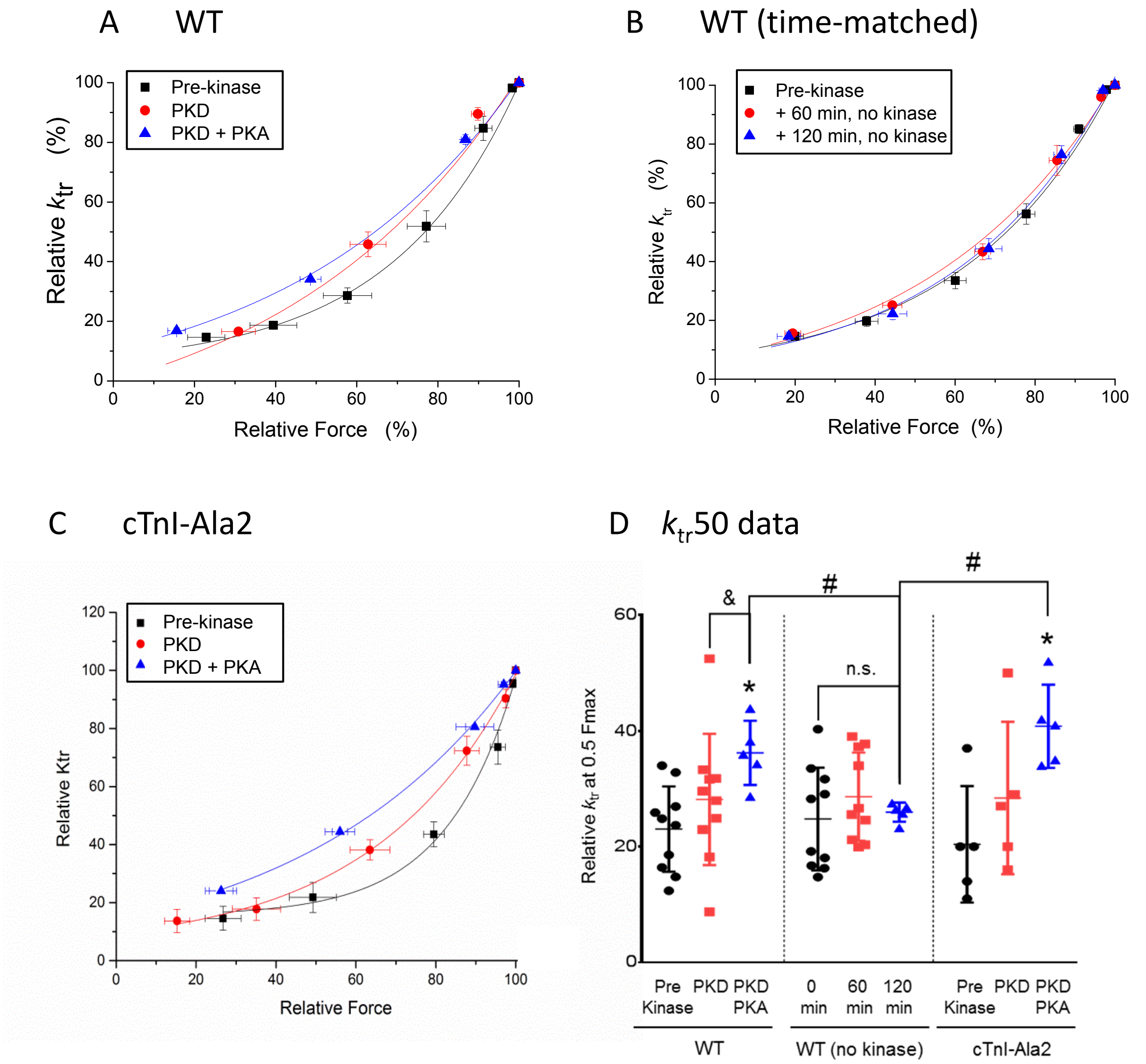

Figure S3. Effects of PKD and PKA incubation on the rate of force redevelopment (k_{tr}) in skinned ventricular trabeculae. Data from the same experiments as shown in main text, Fig. 4. **A,** Measurements of k_{tr} in Ca^{2+}-activated skinned trabeculae from WT mice after incubation in λ-PP (Pre-kinase), PKD and then PKA. Force and k_{tr} values are expressed relative to those recorded at maximum force (30 μmol/L Ca^{2+}). **B**, Time-matched controls for the incubations shown in panel A. **C**, Results using skinned trabeculae from cTnI-Ala2 mice. **D**, Relative k_{tr} values measured at 50% of maximum force. Symbols show the data from individual experiments; vertical bars show the mean ± SD (n=10 for Pre-kinase and PKD incubation groups, n=5 for all other groups).

* $P<0.05$ between Pre-kinase and the corresponding PKD or PKA+PKD incubations
& $P<0.05$ between PKD and PKD+PKA incubations
# $P<0.05$ between PKD incubation and time-matched (PKD) or PKD incubation in cTnI-Ala2 mice
n.s. = non significant.

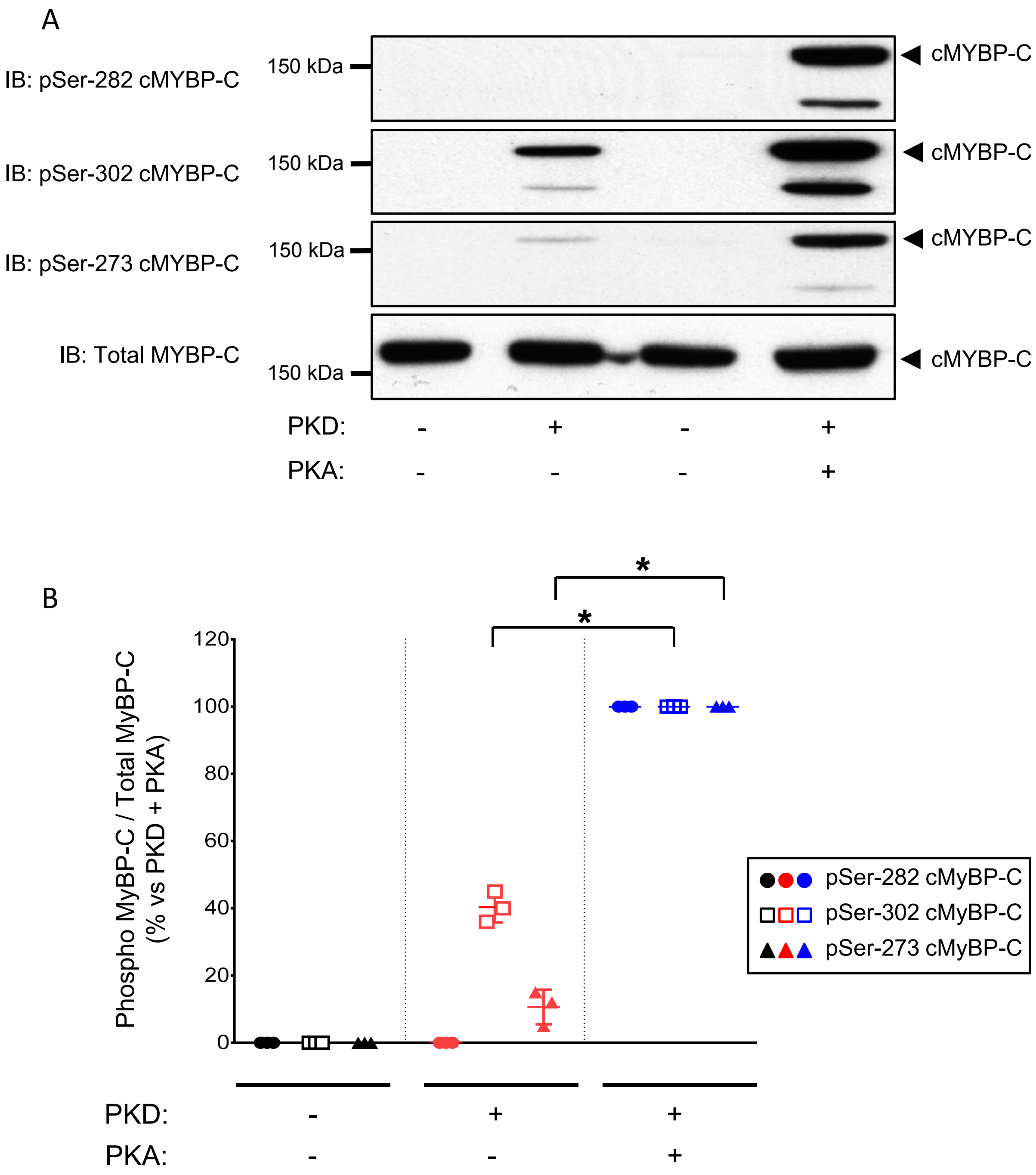

Figure S4. Phosphorylation status of three phospho-serines in cMyBP-C after incubation in PKD alone or PKD followed by PKA. Myofibrils were first dephosphorylated with λ-PP. Equal amounts of the same samples were loaded onto 4 separate gels and run in parallel in the same apparatus. Total MyBP-C and phospho-Ser282, phospho-Ser302 and phospho-Ser273 were detected using specific antibodies (one antibody per gel). **A**, Representative gels. **B**, Collated data. Symbols show the data from individual experiments; vertical bars show the mean ± SD (n=3).

* *P*<0.05 between PKD and PKD+PKA incubations.

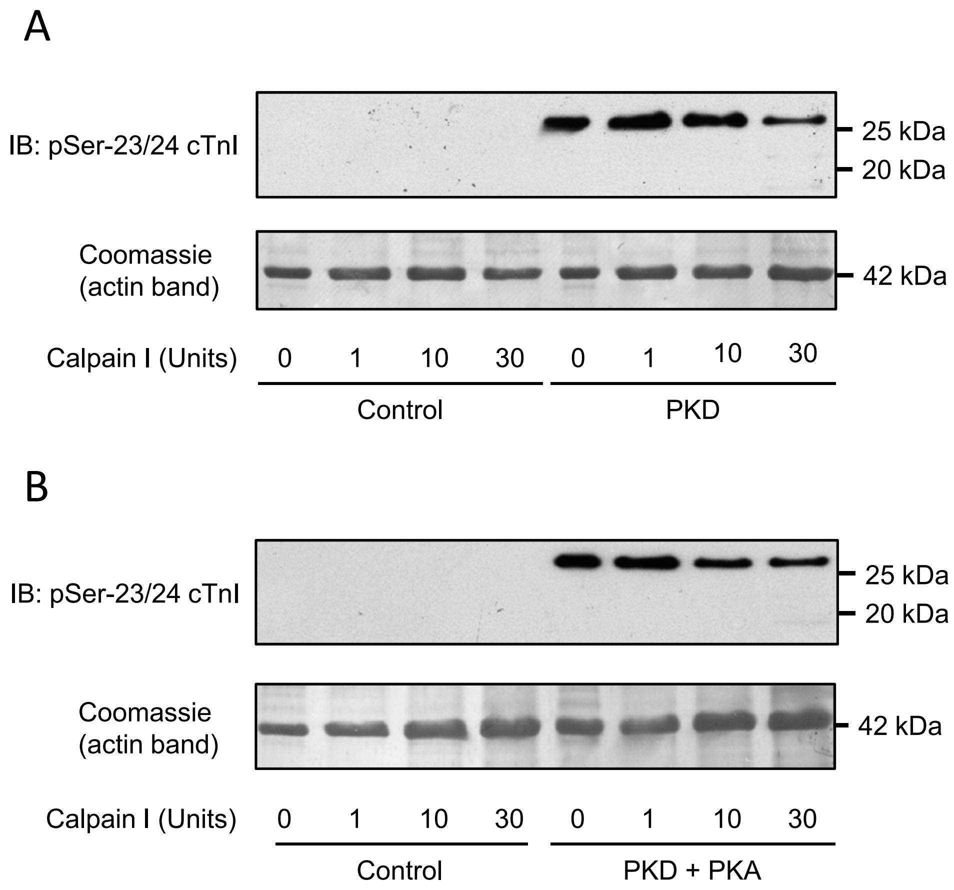

Figure S5: Effect of cTnI mono- and bis-phosphorylation on calpain-induced proteolysis of the C-terminal of sarcomeric cTnI. λ-PP-treated isolated myofibrils were incubated without kinase (Control) or with PKD alone (**A**), or PKD followed by PKA (**B**), then treated with calpain-1 at the activities shown (Units per 100 μL). Upper panels: Western blots using a phospho-specific antibody against Ser23/24 of cTnI. Lower panels: Coomassie staining (loading control). Other details as in main text, Fig 4.

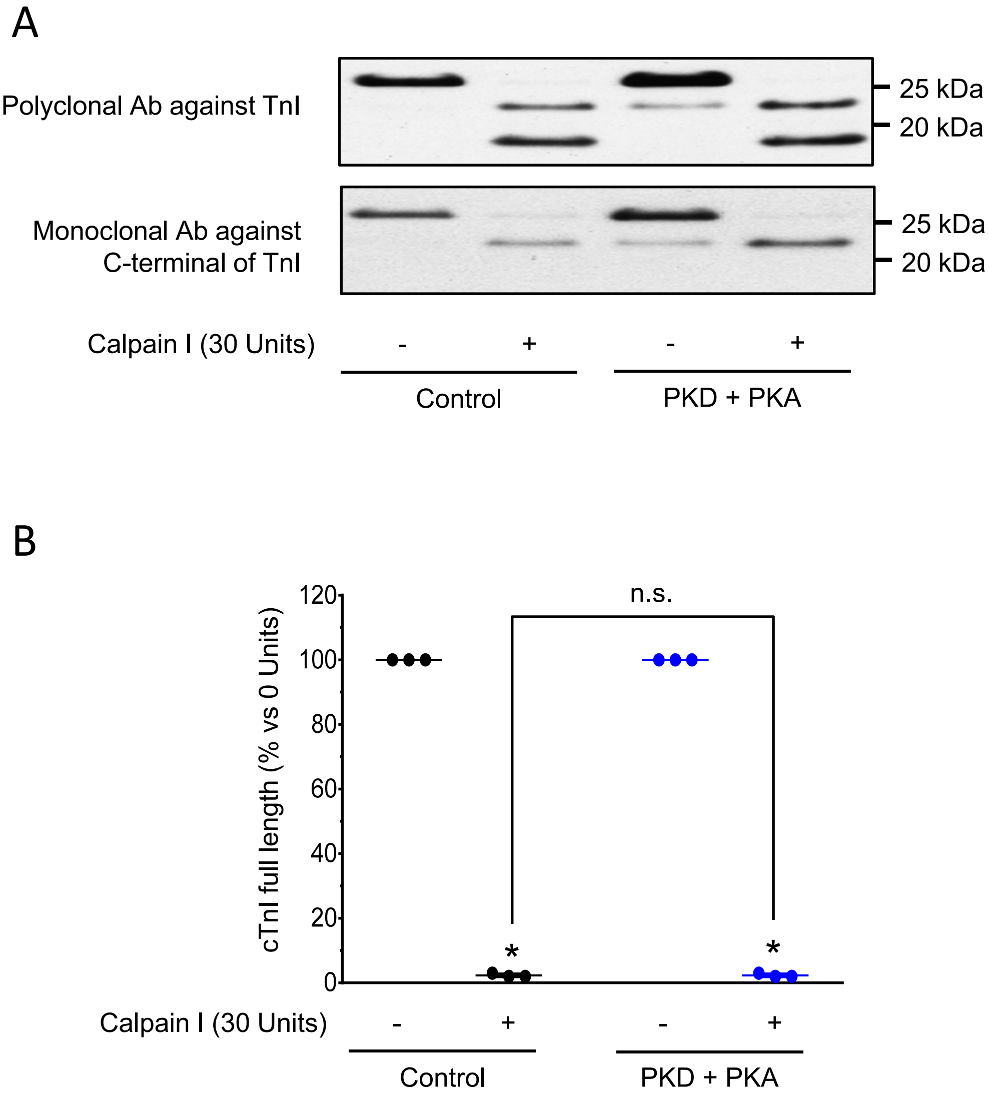

Figure S6: Effect of PKD+PKA incubation on calpain-induced proteolysis of sarcomeric cTnI in myofibrils isolated from cTnI-Ala2 mice. λ-PP-treated isolated myofibrils were incubated without kinase (Control) or with PKD followed by PKA, then treated with calpain-1 (30 U per 100 μL) . **A**, Representative Western blot using an antibody that recognises the core of cTnI or an antibody that recognises the C-terminal of cTnI. **B**, Collated data of TnI degradation, as measured by the optical density of full-length cTnI. Symbols show the data from individual experiments; vertical bars show the mean ± SD (n= 3 for all groups).

* *P*<0.05 vs the respective Control (no-kinase) incubation.
n.s. = non-significant difference (*P*>0.05).

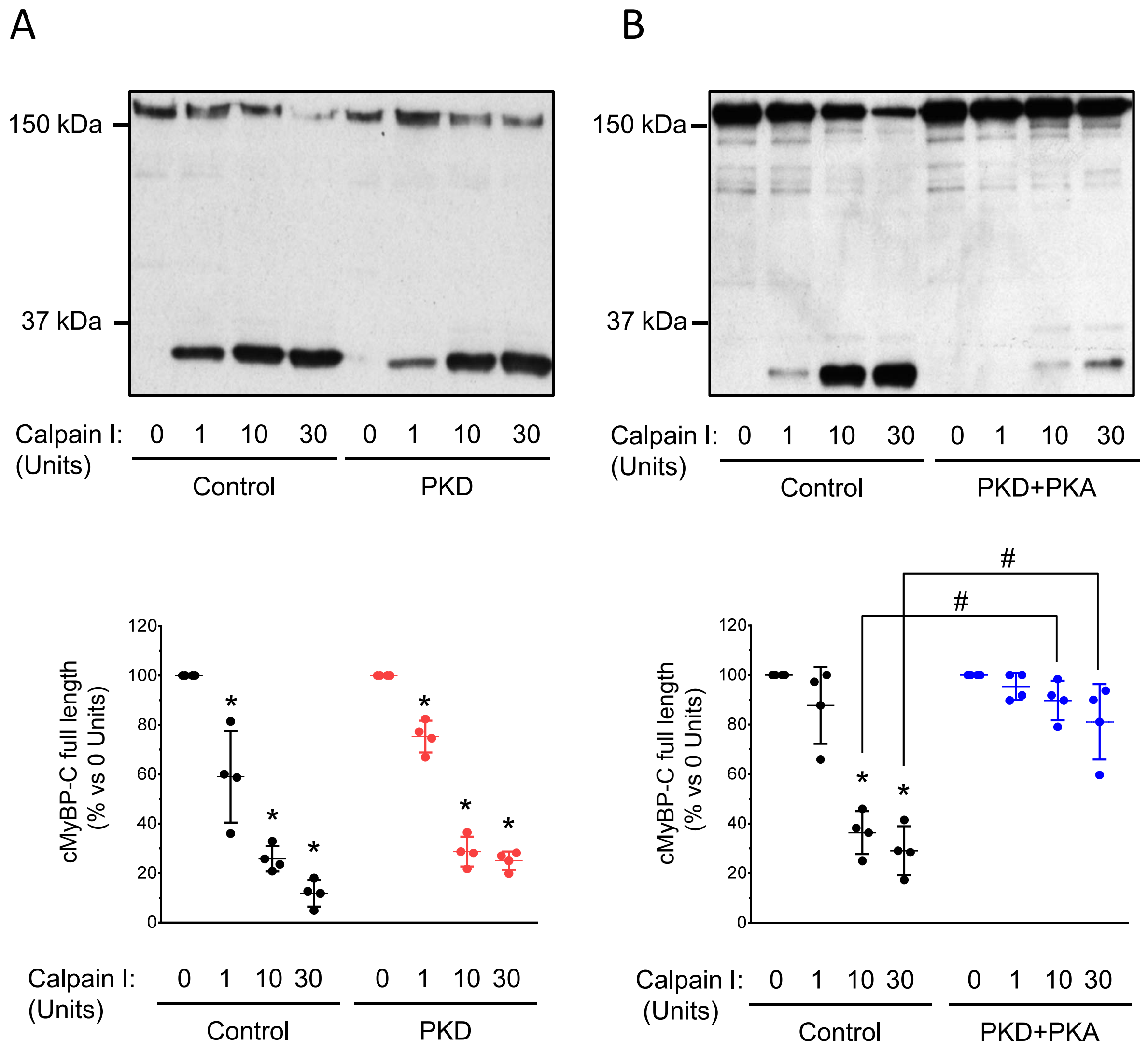

Figure S7. Effect of PKD or PKA incubations on calpain-induced proteolysis of sarcomeric cMyBP-C by calpain. A, λ-PP-treated isolated myofibrils were incubated without kinase (Control) or incubated with PKD, then treated with calpain-1 at the activities shown. Upper panel: representative Western blot using an antibody that recognises the N-terminal of MyBP-C. Lower panel: Collated data of calpain-dependent MyBP-C degradation, measured by the optical density of the full-length cMyBP-C. Symbols show the data from individual experiments; vertical bars show the mean ± SD (n= 4).

* *P*<0.05 vs zero calpain. **B**, As for panel A, but with incubation in PKD followed by PKA. Data are mean ± SEM (n=4). * *P*<0.05 vs zero calpain; # *P*<0.05 Respective control vs PKD+PKA incubation.

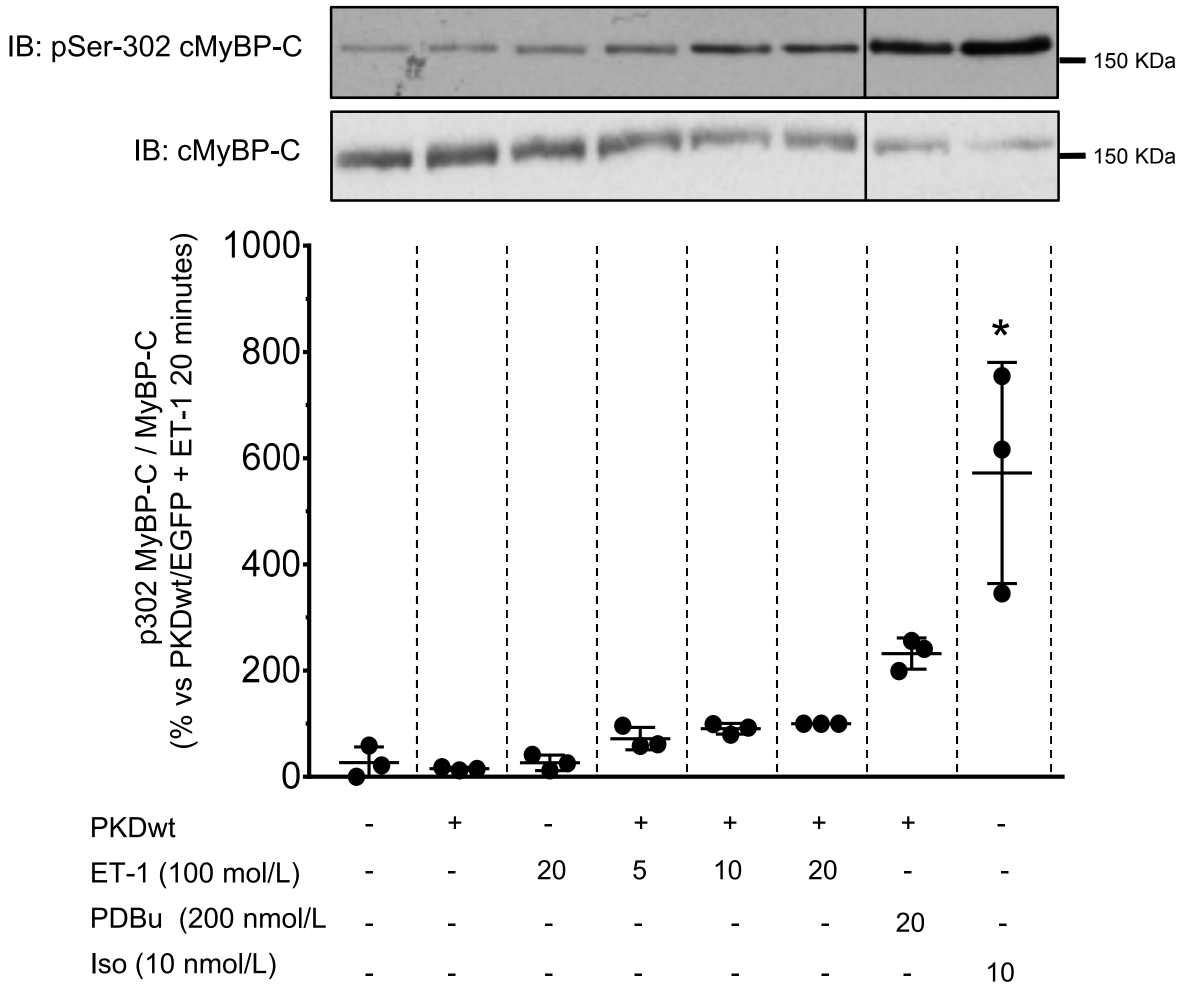

Figure S8. Effect of PKD or PKA activation on cMyBP-C phosphorylation in rat adult cardiomyocytes. Cardiomyocytes infected with adenovirus carrying PKDwt/EGFP (+) or EGFP alone (-) were stimulated with endothelin-1 (ET-1 for 5, 10 or 20 min), PDBu (20 min) or Isoproterenol (Iso, 10 min). These experiments were the same as shown in Fig. 5 of the main text. Upper panel: Representative immunoblots. Equal amounts of the same samples were loaded onto 2 separate gels, were run in parallel in the same apparatus, and probed for phospho-Ser302 cMyBP-C or total cMyBP-C (one antibody per gel). The vertical line shows where an empty lane has been removed by cropping. Lower panel: Collated data. OD of phospho-Ser302 cMyBP-C band / OD of total MyBP-C, normalized to the value for ET-1 (20 min) in PKDwt/EGFP-expressing myocytes. Symbols show the data from individual experiments; vertical bars show the mean ± SD (n=3).

* *P*<0.05 vs PKDwt/EGFP without drugs.

3. Supplemental Table

WT mice	n	Size (µm)	Fmax (mN/mm^2)	Frest (mN/mm^2)	pCa50	EC50 (µM)	Ktr at Fmax (s-1)	Relative Ktr at 0.5 Fmax (%)	Hill Coefficient
Pre-kinase	10	627±40 X 97±7	49.8 ± 4.6	15.5 ± 5.0	6.00 ± 0.04	1.00 ± 0.09	7.53 ± 0.40	23.8 ± 1.4	3.07 ± 0.13
PKD	10	627±40 X 97±8	44.5 ± 4.1	14.0 ± 4.0	5.68 ± 0.03 *#δ	2.08 ± 0.14 *#δ	7.25 ± 0.43*	28.2 ± 3.6	3.27 ± 0.18
PKD (in PKA expts)	5	672±55 X 107±7	39.8 ± 2.9 *	12.9 ± 3.6	5.70 ± 0.01 *#δ	1.93 ± 0.04 *#	7.33 ± 0.41*	25.5 ± 2.5	3.41 ±0.09
PKD+PKA	5	672±55 X 107±7	38.9 ± 3.6 *	12.6 ± 3.6	5.59 ± 0.01 *#δ	2.51 ± 0.08 *#δ	7.12 ± 0.33*	37.5 ± 2.1 *#	3.28 ± 0.03
**WT Time-matched**	**n**	**Size (µm)**	**Fmax (mN/mm^2)**	**Frest (mN/mm^2)**	**pCa50**	**EC50 (µM)**	**Ktr at Fmax (s-1)**	**Relative Ktr at 0.5 Fmax**	**Hill Coefficient**
Pre-, Time-matched	10	653±50 X 97±10	45.0 ± 6.1	14.1 ± 5.0	6.00 ± 0.02	0.98 ± 0.04	7.36 ± 0.30	27.0 ± 2.4	3.14 ± 0.01
+ 60 min, no kinase	10	653±50 X 97±11	39.8 ± 5.3	12.9 ± 4.4	5.92 ± 0.02 *	1.17 ± 0.04 *	7.00 ± 0.28*	30.0 ± 1.8	3.38 ± 0.15
+ 120 min, no kinase	5	650±70 X 84±12	38.8 ± 6.9	12.5 ± 4.8	5.91 ± 0.02 *	1.20 ± 0.06 *	6.43 ± 0.36*	27.8 ± 1.6	3.50 ± 0.14
**cTnI-Ala2 mice**	**n**	**Size (µm)**	**Fmax (mN/mm^2)**	**Frest (mN/mm^2)**	**pCa50**	**EC50 (µM)**	**Ktr at Fmax (s-1)**	**Relative Ktr at 0.5 Fmax**	**Hill Coefficient**
Pre-kinase	5	694±65 X 70±11	52.8 ± 8.6	18.3 ±3.0	5.97 ± 0.02	1.06 ± 0.06	8.01 ± 1.00	20.3 ± 4.5	3.27 ± 0.08
PKD	5	694±65 X 70±11	50.2 ± 5.1	15.6 ± 5.7	5.86 ± 0.02 *	1.33 ± 0.09	6.95 ± 0.93	28.5 ± 5.8	3.24 ± 0.12
PKD+PKA	5	694±65 X 70±11	42.3 ± 4.5*	13.3 ± 2.7	5.85 ± 0.01 *	1.36 ± 0.02 *	6.01 ± 0.72*	40.9 ± 3.3 *#	3.16 ± 0.16

Table S1. Summary data for trabeculae in the time-matched and kinase experimental groups.
Columns are: Number of trabeculae (n); length [between force transducer and motor pins] x diameter (Size); Maximum force divided by cross-sectional area (Fmax); resting force (Frest); Ca^{2+} sensitivity (pCa50); half-maximal effective calcium concentration (EC_{50}); maximum crossbridge cycle kinetics (k_{tr} at Fmax); relative crossbridge cycle kinetics at 50% of maximum force (Relative ktr at 0.5 Fmax); Hill coefficient from sigmoidal fits to the force-pCa data. Data are mean ± SEM.
"PKD (in PKA expts)" shows the data from the 5 PKD experiments in which PKA was subsequently applied to the trabeculae.

* P<0.05 versus Pre-kinase (i.e. λ-PP-treated) data in the same trabeculae (paired t-test).
# P< 0.05 versus the corresponding time-matched value (unpaired t-test), i.e. PKD data were compared with "WT Time-matched +60 min, no kinase", PKD+PKA data were compared with "WT Time-matched, +120 min, no kinase".
δ P<0.05 versus WT PKD or PKD+PKA incubation versus cTnI-Ala2 PKD or PKD+PKA incubation, respectively.

4. References for Supplementary Material

1. Bardswell, S. C., Cuello, F., Rowland, A. J., Sadayappan, S., Robbins, J., Gautel, M., Walker, J. W., Kentish, J. C., and Avkiran, M. (2010) Distinct sarcomeric substrates are responsible for protein kinase D-mediated regulation of cardiac myofilament Ca^{2+} sensitivity and cross-bridge cycling. *J Biol Chem* **285**, 5674-5682
2. Herron, T. J., Korte, F. S., and McDonald, K. S. (2001) Power output is increased after phosphorylation of myofibrillar proteins in rat skinned cardiac myocytes. *Circ Res* **89**, 1184-1190
3. Kulikovskaya, I., McClellan, G. B., Levine, R., and Winegrad, S. (2007) Multiple Forms of Cardiac Myosin-binding Protein C Exist and Can Regulate Thick Filament Stability. *J Gen Physiol* **129**, 419-428
4. Vikhorev, Petr G., Song, W., Wilkinson, R., Copeland, O. N., Messer, Andrew E., Ferenczi, Michael A., and Marston, Steven B. (2014) The Dilated Cardiomyopathy-Causing Mutation ACTC E361G in Cardiac Muscle Myofibrils Specifically Abolishes Modulation of Ca^{2+} Regulation by Phosphorylation of Troponin I. *Biophys J* **107**, 2369-2380
5. Kentish, J. C. (1984) The inhibitory effects of monovalent ions on force development in detergent-skinned ventricular muscle from guinea-pig. *J Physiol* **352**, 353-374
6. Brenner, B., and Eisenberg, E. (1986) Rate of force generation in muscle: correlation with actomyosin ATPase activity in solution. *Proc Natl Acad Sci USA* **83**, 3542-3546
7. Patel, J. R., Fitzsimons, D. P., Buck, S. H., Muthuchamy, M., Wieczorek, D. F., and Moss, R. L. (2001) PKA accelerates rate of force development in murine skinned myocardium expressing α- or β-tropomyosin. *Am J Physiol - Heart & Circ* **280**, H2732-H2739
8. Kawai, M., and Brandt, P. W. (1976) Two rigor states in skinned crayfish single muscle fibers. *J Gen Physiol* **68**, 267-280
9. Cuello, F., Bardswell, S. C., Haworth, R. S., Yin, X., Lutz, S., Wieland, T., Mayr, M., Kentish, J. C., and Avkiran, M. (2007) Protein kinase D selectively targets cardiac troponin I and regulates myofilament Ca^{2+}-sensitivity in ventricular myocytes. *Circ Res* **100**, 864-873
10. Cai, W., Guner, H., Gregorich, Z. R., Chen, A. J., Ayaz-Guner, S., Peng, Y., Valeja, S. G., Liu, X., and Ge, Y. (2016) MASH Suite Pro: A Comprehensive Software Tool for Top-Down Proteomics. *Molecular and Cellular Proteomics* **15**, 703-714
11. Sadayappan, S., Gulick, J., Klevitsky, R., Lorenz, J. N., Sargent, M., Molkentin, J. D., and Robbins, J. (2009) Cardiac myosin binding protein-C phosphorylation in a β-myosin heavy chain background. *Circulation* **119**, 1253-1262
12. Haworth, R. S., Cuello, F., Herron, T. J., Franzen, G., Kentish, J. C., Gautel, M., and Avkiran, M. (2004) Protein kinase D is a novel mediator of cardiac troponin I phosphorylation and regulates myofilament function. *Circ Res* **95**, 1091-1099
